# A live auxotrophic vaccine confers mucosal immunity and protection against lethal pneumonia caused by *Pseudomonas aeruginosa*

**DOI:** 10.1371/journal.ppat.1008311

**Published:** 2020-02-10

**Authors:** Maria P. Cabral, Alexandra Correia, Manuel Vilanova, Fátima Gärtner, Miriam Moscoso, Patricia García, Juan A. Vallejo, Astrid Pérez, Mónica Francisco-Tomé, Víctor Fuentes-Valverde, Germán Bou

**Affiliations:** 1 Department of Microbiology, University Hospital A Coruña (CHUAC)–Biomedical Research Institute A Coruña (INIBIC), A Coruña, Spain; 2 i3S –Instituto de Investigação e Inovação em Saúde, Universidade do Porto, Porto, Portugal; 3 IBMC–Instituto de Biologia Molecular e Celular, Universidade do Porto, Porto, Portugal; 4 Instituto de Ciências Biomédicas Abel Salazar, Universidade do Porto, Porto, Portugal; 5 IPATIMUP—Institute of Molecular Pathology and Immunology, University of Porto, Porto, Portugal; 6 Department of Microbiology, Galicia Sur Health Research Institute (IISGS), Vigo, Spain; Channing Laboratory, Brigham and Women's Hospital, UNITED STATES

## Abstract

*Pseudomonas aeruginosa* is one of the leading causes of nosocomial pneumonia and its associated mortality. Moreover, extensively drug-resistant high-risk clones are globally widespread, presenting a major challenge to the healthcare systems. Despite this, no vaccine is available against this high-concerning pathogen. Here we tested immunogenicity and protective efficacy of an experimental live vaccine against *P*. *aeruginosa* pneumonia, consisting of an auxotrophic strain which lacks the key enzyme involved in D-glutamate biosynthesis, a structural component of the bacterial cell wall. As the amounts of free D-glutamate *in vivo* are trace substances in most cases, blockage of the cell wall synthesis occurs, compromising the growth of this strain, but not its immunogenic properties. Indeed, when delivered intranasally, this vaccine stimulated production of systemic and mucosal antibodies, induced effector memory, central memory and IL-17A-producing CD4^+^ T cells, and recruited neutrophils and mononuclear phagocytes into the airway mucosa. A significant improvement in mice survival after lung infection caused by ExoU-producing PAO1 and PA14 strains was observed. Nearly one third of the mice infected with the XDR high-risk clone ST235 were also protected. These findings highlight the potential of this vaccine for the control of acute pneumonia caused by this bacterial pathogen.

## Author summary

*Pseudomonas aeruginosa* is an opportunistic bacterium and one of the most common causes of healthcare-associated diseases, including acute pneumonia, causing high mortality within immunocompromised hosts. Most of these infections are strikingly difficult to treat using conventional antibiotic therapies, since this microorganism displays high intrinsic resistance to a wide range of antibiotics. Moreover, to date, no vaccine is available for prevention. Here we used a mutated bacterial strain, which is unable to replicate *in vivo* and to cause disease, as a live vaccine against acute pneumonia caused by this pathogen. When applied intranasally, this vaccine induced immunity both at local and distant body sites, activating immune cells which were recruited into the airway mucosa. This evoked immune response reduced the number of non-surviving mice after infection with two cytotoxic *P*. *aeruginosa* strains causing acute lung infection. Some protection was also observed against an internationally disseminated cytotoxic strain. These data indicate that this is a promising vaccine candidate against *P*. *aeruginosa*-pneumonia.

## Introduction

*Pseudomonas aeruginosa* (PA) is one of the most common gram-negative pathogens causing pneumonia in immunocompromised patients[1]. Moreover, the mortality rate of ventilator-associated pneumonia (VAP) due to PA is higher than that due to other pathogens[2]. Acute nosocomial pneumonias due to PA are typically the result of direct trauma, such as damage to the epithelium due to mechanical ventilation in VAP patients[3].

During acute infection, PA secretes exotoxins into the environment that damage host tissue. Namely, type III secretion system (TTSS) works as a molecular syringe, delivering toxins into the cytosol of target eukaryotic cells. ExoU, one of these toxins, disrupts the integrity of the lipid membrane causing epithelial cell damage and lung injury. It also contributes to the release of inflammatory mediators that give rise to inflammation and septic shock. Indeed, high cytotoxicity, severity of lung epithelial injury, and bacterial dissemination into the circulation correlated with the *exoU* genotype in different PA strains[4].

The resistance rates of PA are increasing in many parts of the world: recent studies reported the widespread presence of extreme drug-resistant (XDR) high-risk clones in healthcare settings[5]. Therefore, several evolving translational strategies are being explored for the control and therapy of PA, including immunotherapy[6], phage therapy[7] and vaccination. Several types of vaccines have been developed and tested in Phases I–III clinical trials[8]; however, currently there is no approved option for use in humans.

Respiratory tract infections usually occur or initiate at a mucosal surface like the one lining the lumen of the lung[9]. At the airway surface, inhaled PA become trapped in the viscous mucous layer. Then, flagella, lipopolysaccharide, and type 4 pili are recognized by host pattern recognition receptors such as TLRs thus initiating an inflammatory response[9]. After recognition, activated alveolar macrophages as well as neutrophils recruited by IL-8 phagocytose and kill PA. Dendritic cells (DCs) sample the lung lumen and trigger the adaptive response by presenting antigens to T cells. Therefore, vaccine-based immunity against PA should ideally induce local immunity at the lung mucosa.

Mucosal vaccination has superior ability to induce local mucosal immune responses over systemic vaccination[10]. In addition, immunization at one mucosal site can result in antibody secretion systemically, as well as at other selected mucosal sites[10]. These features can prevent the establishment and dissemination of an infection, particularly if it initiates at a mucosal surface.

We previously characterized an experimental live vaccine against PA (α-PA vaccine), composed of a D-glutamate (D-Glu) auxotrophic strain with inactivated Glutamate racemase (MurI), a key enzyme that provides the D-Glu required for peptidoglycan biosynthesis[11]. This live vaccine has a self-limited growth *in vivo*, elicits functional and cross-reactive antibodies, triggers cellular immunity and confers protection against disease caused by different PA strains in a murine sepsis model. The robust protection observed in immunized mice–~100% vaccine efficacy–, and the so far scarce description of effective prototype vaccines prompted us to evaluate whether this D-Glu auxotroph holds the potential to be immunogenic and effective against lethal pneumonia caused by PA.

In the present study, we explored a mucosal vaccination route to deliver our live α-PA vaccine, having found that it elicits a multifactorial immunity, involving T cells and systemically and mucosally-produced antibodies. This multifactorial immunity was correlated with partial, but considerable protection against acute fatal lung infection caused by two highly virulent ExoU-positive cytotoxic PA strains, and mild protection against a XDR disseminated clone, also ExoU-positive. Some constrains posed by the mild dose-dependent toxic effect observed, prompted us to test parenteral routes for immunization also, and their effect on mucosal immunity against acute pneumonia. Intramuscular, intradermal and subcutaneous immunization conferred significant improvement in survival of PA14-infected mice, however, transient adverse reactions were observed with the last two. Intramuscular immunization conferred only moderate protection, however, when applied in combination with intranasal route, it allowed using lower and safer doses of vaccine intranasally resulting in considerable efficacy.

We conclude that our D-Glu auxotrophic strain enables local, as well as systemic immunity providing heterogeneous but considerable protection against lethal pneumonia caused by cytotoxin-producing strains, thus it should be considered as a promising prototype vaccine against PA in the future.

## Results

### Characterization of α-PA vaccine-induced antibody responses after intranasal immunization

**Fig 1:**
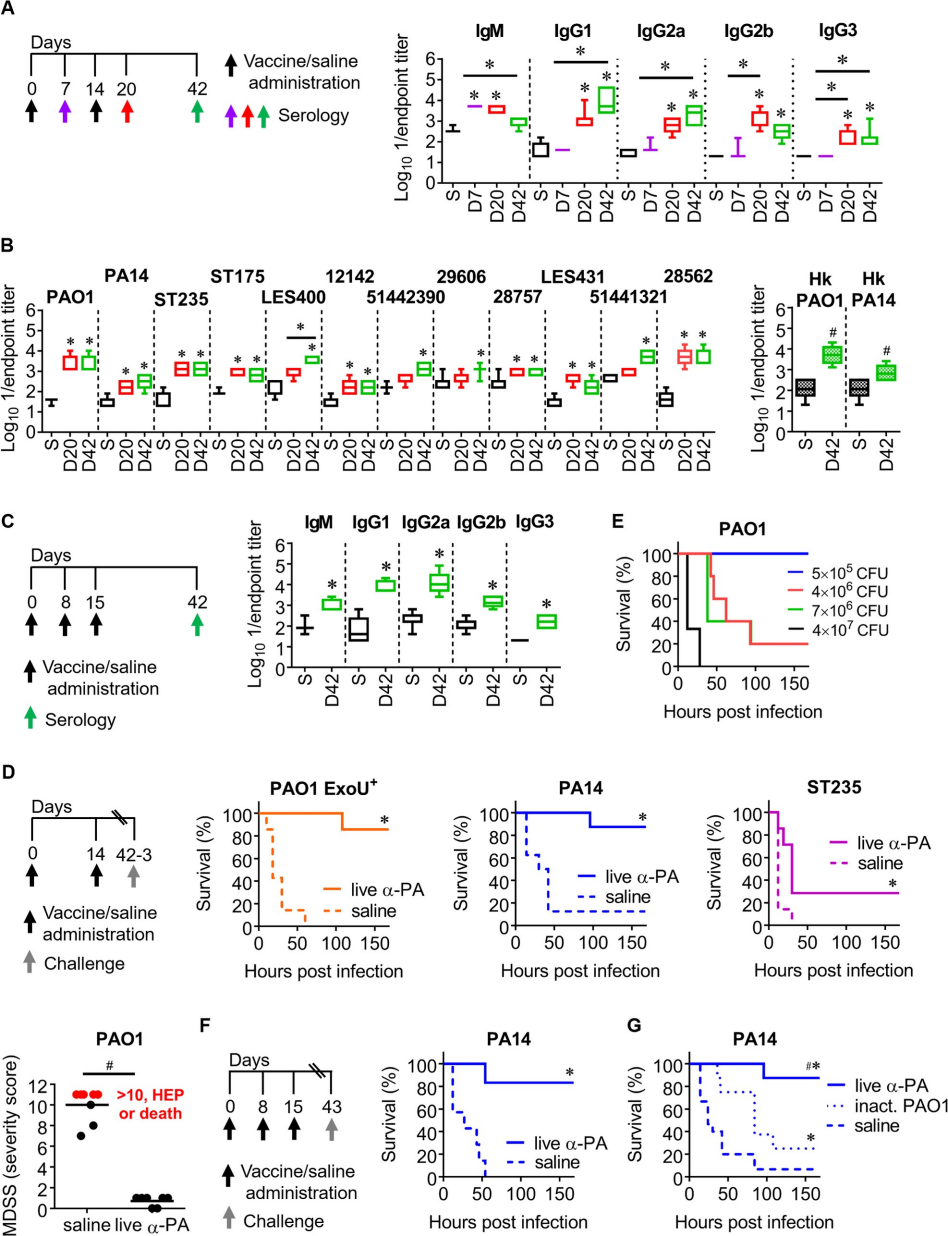
α-PA vaccine generates cross-reactive antibodies and heterogeneous protection against PA-pneumonia. **(A)** Immunization schedule and antibody titers against PAO1 in vaccinated (*n* = 7) and control (*n* = 7) mice after one or two IN immunizations with α-PA vaccine (2×10^8^ CFU), or saline administration, respectively. **(B)** IgG titers against different live and Hk PA strains in vaccinated (*n* = 6–7) and control (*n* = 5–7) mice after two IN immunizations with α-PA vaccine (2×10^8^ CFU), or saline administration, respectively. **(C)** Immunization schedule and antibody titers against PAO1 in vaccinated (*n* = 5) and control (*n* = 6–7) mice after three IN immunizations with α-PA vaccine (2×10^8^ CFU), or saline administration, respectively. **(A-C)** S, saline; D, day. **(A, B)** *P<0.05 (Kruskal-Wallis test), compared with saline and between the indicated groups. **(B)** ^#^*P*<0.05 (Mann-Whitney U test), compared with saline group. **(C)** *P<0.05 (Mann-Whitney U test), compared with saline group. **(D)** Immunization schedule, mice survival and assessment of murine disease severity score (MDSS) after IN administration of α-PA vaccine (2×10^8^ CFU), or saline and IN challenge with PAO1 ExoU^+^ (7×10^5^ CFU, *n* = 7), PA14 (1×10^6^ CFU, *n* = 8), ST235 (3×10^7^ CFU, *n* = 7) and PAO1 (2×10^6^ CFU, *n* = 7–8). **(E)** Mice survival after IN challenge with different doses of PAO1 (*n* = 3–5 mice/group). **(F)** Immunization schedule and mice survival after IN administration of α-PA vaccine (2×10^8^ CFU, *n* = 6), or saline (*n* = 7) and IN challenge with PA14 (1×10^6^ CFU). **(G)** Mice survival after IN immunization with α-PA vaccine (2×10^8^ CFU, *n* = 8), inactivated PAO1 (2×10^8^ CFU, *n* = 8) or saline administration (*n* = 15) and IN challenge with PA14 (1×10^6^ CFU), according to schedule **(D)**. **(D, F and G)** *P<0.05 (log-rank test), compared with saline group. **(D)** #*P*<0.05 (Mann-Whitney U test). **(G)** #*P*<0.05 (log-rank test), compared with mice administered inactivated PAO1.

PA PAO1 was previously genetically manipulated to obtain a D-Glu auxotrophic strain, PAO1 Δ*murI*, by eliminating the MurI coding gene, *murI*[11]. Mice were inoculated intranasally (IN) with PAO1 Δ*murI* (2×10^8^ CFU)–live α-PA vaccine–on days 0 and 14. An extra immunization was performed on day 8, when using a three-dose schedule. Control mice were inoculated saline. PAO1-specific antibody levels were determined in sera obtained at different time points. When using the two-dose schedule (Fig 1A), significantly elevated levels of IgM were present on day 7, while increased IgG1, IgG2a, IgG2b and IgG3 titers were detected on days 20 and 42. IgM levels were significantly reduced on day 42, indicating isotype switching.

Cross-reactivity of the total IgG raised by using the two-dose schedule was determined against 11 heterologous PA strains (S1 Table and Fig 1B). The levels of anti- PA14 (ExoU-producing strain), -ST235 (XDR high-risk clone), -ST175 (XDR high-risk clone), -LES400 and -12142 (epidemic strains from CF patients), -51442390 (Mem^R^ mucoid isolate from a CF patient), -29606 and -28757 (mucoid isolates from CF patients), -LES431 (epidemic strain from a non-CF patient), -51441321 and -28562 (MDR/mucoid isolates from bronchiectasis patients) IgG’s were significantly elevated on day 42, compared with the saline group. The mucoid phenotype is associated with chronic infections from cystic fibrosis patients[12]. When using a three-dose schedule, IN vaccination also resulted in significantly elevated PAO1-specific antibody levels of all assessed isotypes (Fig 1C). IgG titers against boiled PAO1 and PA14 (which should destroy heat-labile protein epitopes) (Fig 1B) were similar to those recognizing whole bacteria, indicating that IgG antibodies raised by immunization mostly recognize heat-stable antigens, which might include O antigen.

### Assessing protective efficacy against acute lung infection using α-PA IN vaccination

We assessed the survival of mice IN-challenged with three cytotoxin-producing PA strains causative of acute lung infection for testing the cross-protection of the α-PA vaccine and the robustness of the pneumonia model (Fig 1D).

PAO1 ExoU^+^ is a derivative from PAO1 carrying *exoU* gene. Introduction of this gene in PAO1 confers a cytotoxic phenotype and increased virulence in a murine model of acute pneumonia and systemic spread[13]. PA14 is a highly cytotoxic *exoU*^+^ reference strain[14,15] representing the most common clonal group worldwide[16]. PA ST235 is an international XDR high-risk clone, which is among the most disseminated XDR clones in hospitals worldwide[17]. ST111 and ST175 are likely the other more widespread ones[5,18,19]. Here we used the previously described ST235 strain 8[15], since it is able to cause mortality in a peritonitis/sepsis model in mice, in contrast to ST111 and ST175. Moreover, ST235 contains the *exoU*-encoded exotoxin[17].

Survival rates of vaccinated mice using a two-dose vaccination schedule were 86, 88 and 29% after challenge with PAO1 ExoU^+^, PA14 and ST235, respectively; whereas control mice presented 0, 13 and 0% survival (Fig 1D). Despite this heterogeneous protection, the differences in survival were significant for the three assays, indicating a general reduction in the severity of disease in vaccinated animals.

We also tested vaccine efficacy against the wild-type PAO1, as ExoU-negative strains typically require higher challenge doses to cause 100% lethality, thus giving a better assessment of the potency of the vaccine. Indeed, whereas inoculation of 7×10^5^ CFU of PAO1 ExoU^+^ caused 100% mortality (Fig 1D), challenge doses as high as 4×10^7^ CFU of PAO1 were required to observe 100% death (Fig 1E). But because infected mice showed indicators of pain, suffering and illness, a murine disease severity score (MDSS) was used for monitoring surrogate endpoints and determining disease severity after challenge with a lower dose. Five criteria were assessed to provide an overall score between 1 and 10, which subsequently dictated the clinical condition and/or euthanasia (HEP, human endpoint) (S2 Table). Succumbed mice and HEP were given a score of 11. Compared to vaccinated mice, sham-immunized mice had a significantly higher mean score (0.71 *vs* 10, respectively) (Fig 1D). Moreover, 2 out of 8 control mice died, whereas 3 presented scores compatible for HEP, thus indicating a reduction in the severity of infection. Using a three-dose immunization schedule, survival rates of vaccinated mice were 83%, after challenge with PA14, whereas all control mice died (Fig 1F). Considering that using an extra immunization dose did not boost the protective effect of the vaccine substantially, we kept the two-dose schedule for the posterior IN immunizations. To further investigate whether our auxotrophic strain has any benefit as a vaccine over inactivated bacteria, we compared its protective efficacy with formalin-inactivated PAO1. As shown in Fig 1G, only one out of 8 mice administered α-PA vaccine and infected with PA14 died, whereas 93% of control mice succumbed to acute pneumonia within 84 h. In contrast with live vaccine, 75% of mice administered formalin-inactivated PAO1 succumbed to infection. The protection conferred by α-PA vaccine was significantly higher when compared with formalin-inactivated PAO1. These results show that IN immunization with α-PA vaccine offers an advantage over wild-type inactivated bacteria in the improvement of survival.

### Cellular immune response induced by α-PA IN vaccination before and after infection

**Fig 2:**
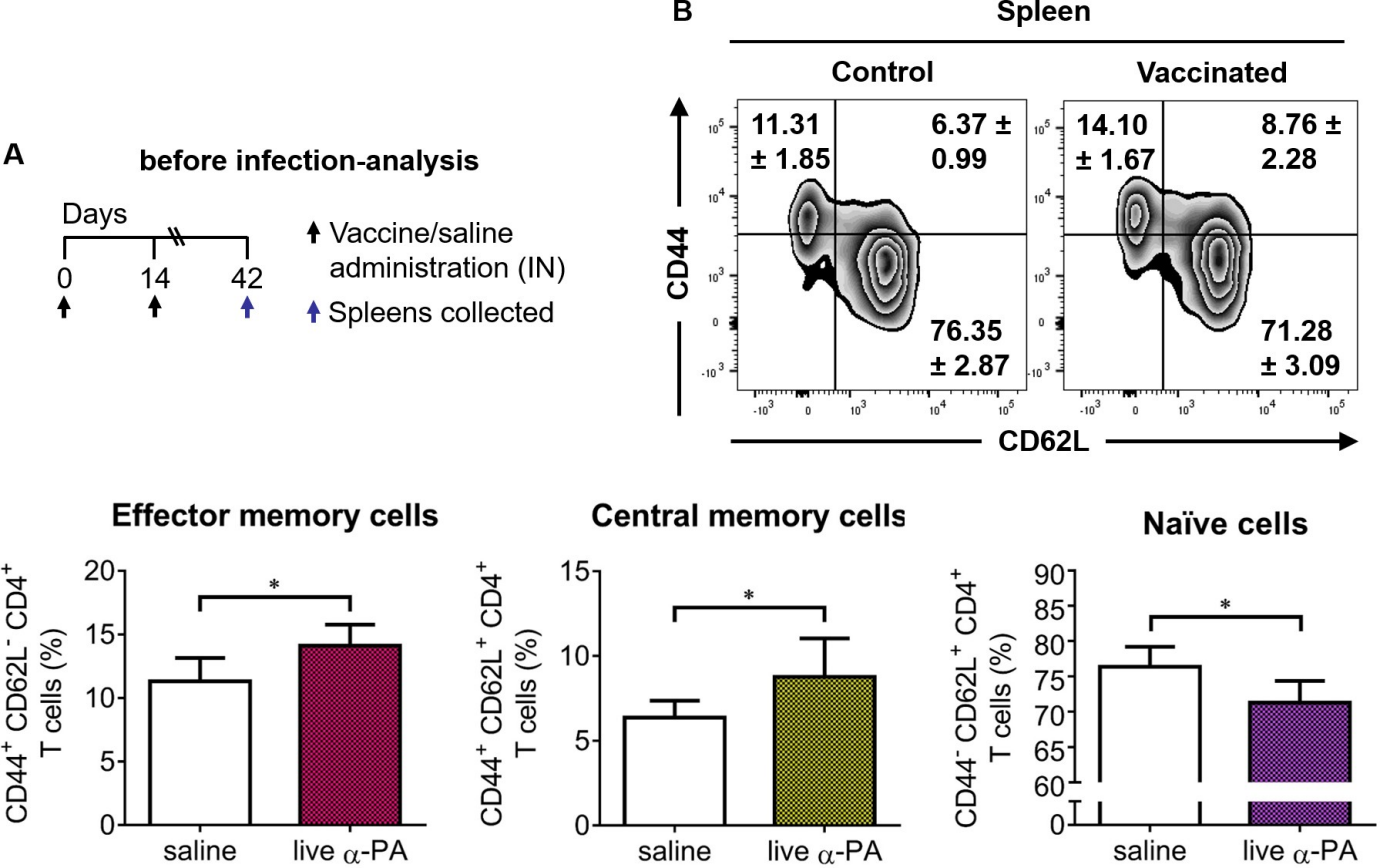
Production of memory CD4^+^ T cells in vaccinated mice. **(A)** Mice (*n* = 6/group) were immunized with α-PA vaccine (2×10^8^ CFU) or administered saline, according to the schedule; then spleens were collected. **(B)** Percentage of spleen effector memory (CD44^+^ CD62L^-^), central memory (CD44^+^ CD62L^+^) and naïve (CD44^-^ CD62L^+^) CD4^+^ T cells. Contour plots correspond to a representative example of CD4-gated T cells of the analyzed samples. Analysis regions were set according to FMO and isotype control-stained samples. Numbers inside plot regions represent means ± SD of the frequency of cells due to respective cell staining. Bars represent mean ± SD of data. *P<0.05 (t-test).

As shown in Fig 2, the proportion of CD4^+^ T cells displaying the CD44^+^CD62L^-^ and CD44^+^CD62L^+^ phenotypes were slightly but significantly increased in the spleens of α-PA vaccinated mice as compared to controls, which respectively indicates the stimulation of effector and central memory CD4^+^ T cells. The gating strategy used to discriminate memory cells by flow cytometry is presented in S1 Fig.

**Fig 3:**
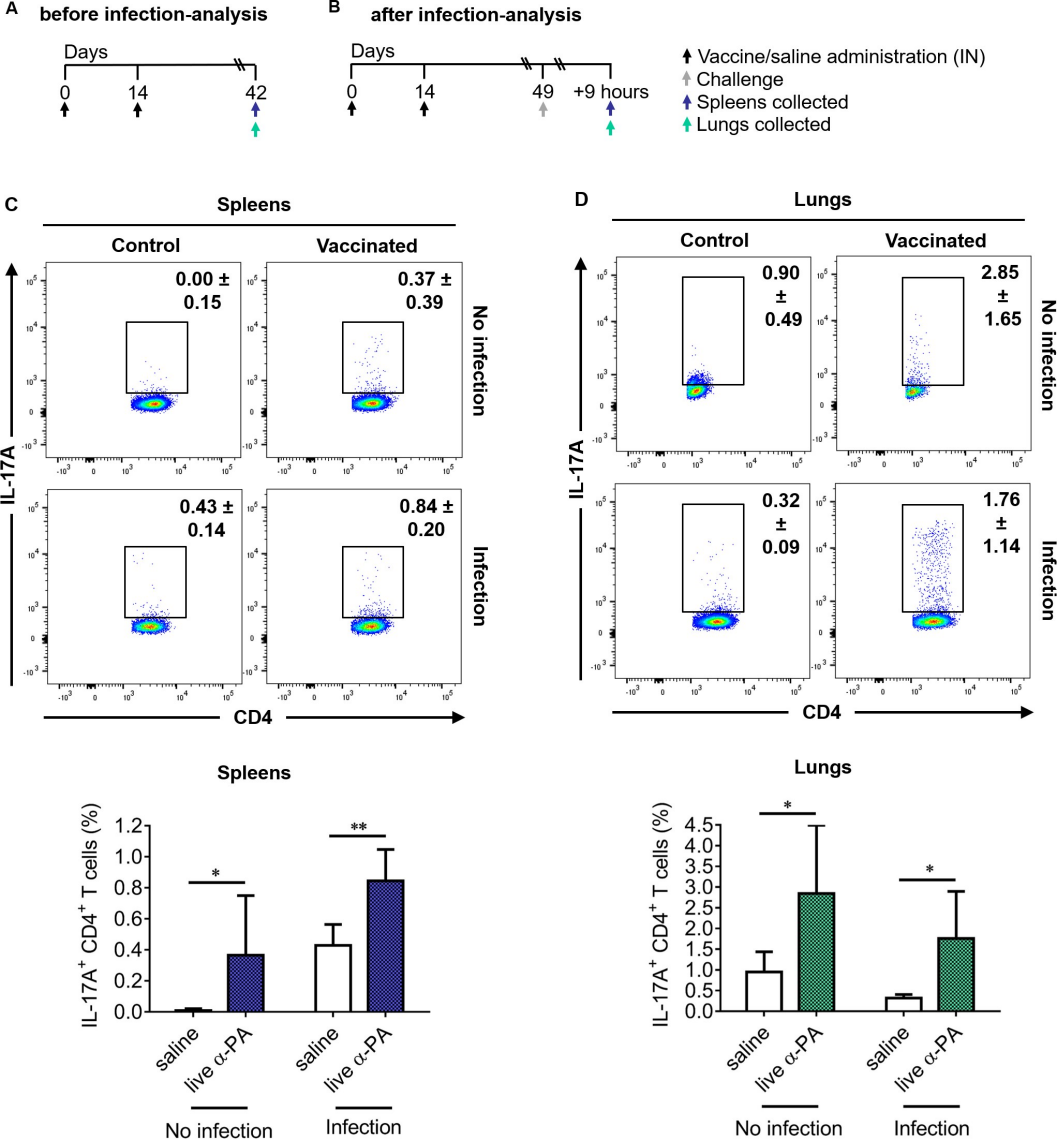
Increased production of IL-17A by CD4^+^ T cells of vaccinated mice. **(A, B)** Mice (*n* = 6/group) were immunized with α-PA vaccine (2×10^8^ CFU) or administered saline, according to the schedule; then spleens and lungs were collected. In **(B),** mice (*n* = 6/group) were infected with PA14 (1×10^6^ CFU), 9 h before the spleens and lungs were obtained. **(C, D)** Representative examples and percentage of CD4-gated T cells expressing IL-17A obtained from spleens **(C)** and lungs **(D)** of infected and non-infected mice, detected by intracellular staining after stimulation with PMA/ionomycin. Analysis regions were set according to FMO and isotype control-stained samples. Numbers inside dot plot regions represent means ± SD of the frequency of cells due to respective cytokine staining. Bars represent mean ± SD of data. *P<0.05, **P<0.01 (t-test).

To better elucidate the effector function of CD4^+^ T cells induced by vaccination, the proportions of splenic and lung CD4^+^ T cells producing IL-17A (Fig 3), IFN-γ, IL-10, IL-4 and TNF-α (S2 Fig) was determined by flow cytometry analysis in both infected and non-infected mice. Representative dot plots showing different cellular populations are presented in Fig 3 and S3 and S4 Figs. The corresponding gating strategy is presented in S5 Fig. An increased frequency of splenic CD4^+^ T cells expressing IL-17A was observed for vaccinated mice compared to controls, both in infected and non-infected mice (Fig 3C). The same was observed for CD4^+^ T cells expressing IL-17A from lungs (Fig 3D). The frequencies of splenic CD4^+^ T cells expressing IFN-γ, IL-10 and TNF-α were found similar in vaccinated and control mice, whereas a reduction of CD4^+^IL-4^+^ cells frequency was observed upon infection in vaccinated mice (S2 Fig). In the lungs, no different proportions of cells producing any of these cytokines were found between vaccinated mice and controls, except for a decreased frequency of CD4^+^ T cells expressing TNF-α in non-infected vaccinated mice (S2 Fig). γδ T cells have also been shown to be a potent source of IL-17 and, in some cases, to produce more IL-17 than αβ T cells[20]. As shown in S6 Fig, a considerable increase of IL-17A^+^ γδ T cells was detected in the lungs upon infection, that was however not different between vaccinated and control mouse groups. Also, no significant differences in IFN-γ^+^ γδ T cells were observed between vaccinated and control mice (the gating strategy is shown in S7 Fig). This suggests that protection conferred by our vaccine is not dependent on these T cell populations.

To confirm the type of T cell response generated by the α-PA vaccine, IFN-γ, IL-17A, IL-10 and IL-4 levels were evaluated in culture supernatants of antigen-stimulated splenocytes obtained from vaccinated and non-vaccinated mice prior to and after being infected with PA14. As shown in S8 Fig, splenocytes recovered from vaccinated-infected mice responded to heat-killed (Hk) PA14 by producing significantly higher amounts of pro-inflammatory cytokines IL-17A and IFN-γ than sham-immunized controls. Nevertheless, the results from splenocyte cultures of non-infected mice indicate that immunization preferentially induces commitment of responding cells for IL-17A production, as the PA14 antigen induced IFN-γ production also in cells from non-immunized animals. Higher production of counterinflammatory cytokine IL-10 was also observed for cells obtained from vaccinated mice, however, at lower amounts than IL-17A and IFN-γ. IL-4 was not detected in any assessed condition.

These results indicate that α-PA vaccine triggers a cellular immune response polarized to the production of IL-17A in spleens and lungs, suggesting that this cytokine mediates the protective effect of the vaccine against the acute lung infectious process.

Bacteria clearance requires phagocyte activity and neutrophils and other phagocytes are essential innate cellular effectors for immunity against PA-pneumonia[9,21–23]. Thus, the numbers of myeloid cells of different populations were determined in the lungs of α-PA vaccinated and control mice, before and after PA infection, using flow cytometry (S9 Fig). Neutrophils, eosinophils, alveolar macrophages (AM), recruited monocytes and dendritic cell (DC) populations were defined as shown in the gating strategy presented in S10 Fig. IN vaccination with α-PA led to a significant increase in the number of lung DCs of both CD11b^-^ and CD11b^+^ subsets and of Ly6C^-^ Monocytes/Macrophages (Mo/Mφ). CD11b^+^ increase is especially relevant as they include professional antigen presenting cells (APCs). Upon infection, the numbers of CD11b^+^ DC in vaccinated mice remained above controls. Ly6C^-^ Monocytes/Macrophages (Mo/Mφ), often recognised as resident monocytes that are the precursors of tissue macrophages[24], were still elevated upon infection. Alveolar macrophages and neutrophils were also found at increased numbers in infected-vaccinated mice when compared to controls. These results suggest that vaccination increased the recruitment/proliferation of mononuclear and polymorphonuclear cells into the lungs, which might contribute to more bacterial clearance.

### Intramuscular (IM) immunization with α-PA vaccine stimulates humoral immunity but offers reduced protection against infection

**Fig 4:**
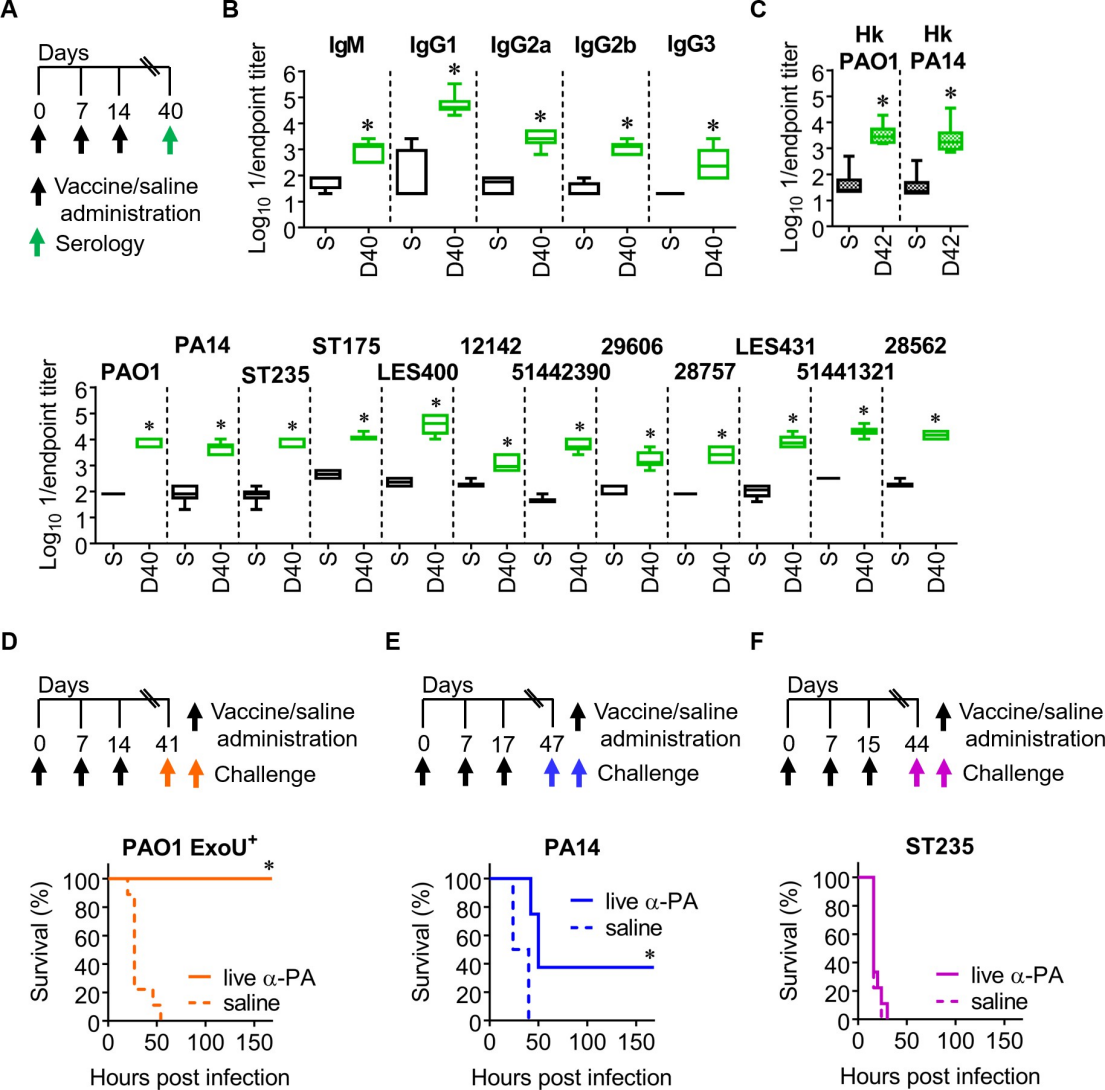
IM immunization with α-PA vaccine generates systemic and cross-reactive antibodies but reduced protection against pneumonia. **(A)** Immunization schedule for **(B)** and **(C). (B)** Antibody titers against PAO1 in vaccinated (*n* = 6) and control (*n* = 6) mice after IM immunization with α-PA vaccine (3×10^7^ CFU), or saline administration, respectively. **(C)** IgG titers against different live and Hk PA strains in vaccinated (*n* = 6) and control (*n* = 6) mice after IM immunization with α-PA vaccine (3×10^7^ CFU), or saline administration, respectively. **(B, C)** S, saline; D, day. *P<0.05 (Mann-Whitney U test), compared with saline group. **(D)** Immunization schedule and mice survival after IM immunization with α-PA vaccine (3×10^7^ CFU, *n* = 8), or saline administration (*n* = 9) and challenge with PAO1 ExoU^+^ (1×10^5^ CFU). **(E)** Immunization schedule and mice survival after IM immunization with α-PA vaccine (3×10^7^ CFU, *n* = 8), or saline administration (*n* = 8) and challenge with PA14 (9×10^5^ CFU). **(F)** Immunization schedule and mice survival after IM immunization with α-PA vaccine (3×10^7^ CFU, *n* = 9), or saline administration (*n* = 9) and challenge with ST235 (5×10^6^ CFU). **(D-F)** *P<0.05 (log-rank test).

Because different vaccine candidates against PA infections have been tested in clinical trials using IM administration[25,26] we tested the immune response generated with α-PA vaccine using this route. Mice were IM-inoculated with α-PA vaccine (3×10^7^ CFU) on days 0, 7 and 14. Control mice were sham-immunized with saline. To assess the humoral response elicited by the immunization, the levels of PAO1-specific IgM and IgG isotypes were determined in sera (Fig 4A). The cross-reactivity of the IgG obtained after finalizing the vaccination schedule was also determined as before. Serum antibody levels were comparable to those obtained when using the IN route on day 42 (Figs 4B and 1A), with the exception of IgM, which remained elevated after IM immunization. IM immunization also resulted in increased anti-PA IgG1, IgG2a, IgG2b and IgG3 levels. A similar trend was seen for the cross-reactivity of serum IgG obtained and for antibodies recognizing heat-stable antigens (Fig 4C). Thus, it is unlikely that different immunization routes may induce IgG antibodies with clearly different specificities, such as protein *vs* carbohydrate antigens.

We next evaluated whether the immune response induced by IM immunization reduces the severity of disease at the same level as IN immunization. Although different vaccination schedules were used for IN and IM delivery, we established this comparison because antibody levels and cross-reactivity obtained was similar between them. After challenging mice with PAO1 ExoU^+^, IM vaccination resulted in 100% survival (Fig 4D). This result was superior to that obtained with IN immunization (86% survival, Fig 1D). However, when the challenge was performed with PA14, survival of IM-vaccinated mice was 38% (Fig 4E), contrasting with the 88% survival observed for IN-vaccinated mice (Fig 1D). Finally, when using the ST235 strain, all mice receiving the IM-α-PA vaccine did not survive lethal challenge (Fig 4F), in contrast with the 29% survival presented by IN-vaccinated mice (Fig 1D). This suggests that α-PA vaccine is less effective against pneumonia caused by cytotoxin-producing strains when applied through IM route, offering reduced protection.

To elucidate whether the lower protection against the LPS-heterologous strain PA14 after IM immunization could be related with the lack of cellular immune responses, we performed flow cytometry analysis and cytokine quantification in the culture supernatants as previously made for IN-vaccinated mice. In contrast to IN-immunized mice, non-infected IM-vaccinated mice presented no increased numbers of effector and central memory CD4^+^ T cells, thus indicating that this route of immunization does not stimulate an adaptive response at the same level as the IN-route (S11 Fig). In the spleens of IM-immunized mice, the frequencies of CD4^+^ T cells expressing IL-17A, IFN-γ, IL-10 and TNF-α were found similar when compared to controls, both before and after infection. The only exception was a significant increased frequency of CD4^+^ IL-4^+^ T cells in non-infected mice. In the lungs, no differences in CD4^+^ T cells populations were found (S12 Fig). On this occasion, none of the assessed cytokines were significantly produced by IM-vaccinated mice, except for IL-17A (S13 Fig). These IL-17A levels, however, were much lower when compared to IN-vaccination (mean 127 *vs* 2489 pg/mL, before infection; mean 422 *vs* 4782 pg/mL, after infection; 1:1 stimuli). In contrast to IN vaccination, no significant increase in the number of lung DCs (neither CD11b^-^ nor CD11b^+^ subsets) was observed after IM immunization (S14 Fig). This might support the observation that T cell differentiation was essentially not different between IM-vaccinated and control mice. Moreover, the increase in Ly6C^-^ Monocytes/Macrophages (Mo/Mφ) was modest when compared to IN vaccination (S14 Fig, panels A and C). Somehow surprisingly, Ly6C^+^ Monocytes/Macrophages (Mo/Mφ) recruitment was stimulated after IM immunization, but not after IN immunization. Although Ly6C^+^ monocytes might differentiate into Ly6C^-^ monocytes or moDCs, no increased numbers of cells of such populations were detected upon infection. Upon infection, a significantly higher number of Ly6C^-^ Mo/Mφ and neutrophils were found in IM-vaccinated mice when compared to controls, as previously observed in IN-vaccinated mice, but not of alveolar macrophages (S14 Fig, panels B and D). In short, these results might indicate that IM immunization with live α-PA vaccine does not promote DCs accumulation in the lungs at the same level as IN vaccination, and suggest a minor effect of this immunization route in the numbers of lung myeloid populations.

We also compared the different specificities of the antisera from IN- and IM-immunized mice against PA14, using passive immunization experiments. However, mice given IgG-enriched anti-PA serum from immunized donors presented no significant differences in survival when compared with mice given naïve serum (0.3% *vs* 0.2% *vs* 0.2% survival, respectively) (S15 Fig). These results suggest that the improvement in survival conferred by IN vaccination is not dependent on serum IgG antibodies and thus the lower protection observed with the IM route cannot be associated with different specificities of the systemic response.

### Safety assessment of α-PA vaccine

α-PA vaccine or vehicle (saline) were administered IN and IM to mice, approximately according to the previous regimens and schedules. As clinical toxicological signs can be difficult to discern by visual examination, we recorded mice body weight prior to and during the immunization regimens (S16 Fig). When using the IN route (2×10^8^ CFU), a mean 13% weight loss was observed after the first administration of α-PA vaccine, on day 2. After the second administration (day 16), mice presented 15% weight loss. These differences were significant when using mixed ANOVA with “study day” as within-subjects factor. Althought significant weight differences with respect to controls were observed until completion of the experiment, these animals recovered their body weight at pre-treatment on days 8 and 22 (S16A Fig). In contrast to IN immunization, no body weight loss was observed for IM-vaccinated mice (S16B Fig).

Considering that IM immunization was performed using a lower vaccine dose (3×10^7^ CFU), we tested the same dosing regimen, but using the IN route (S16C Fig). On this occasion, significant weight loss was only seen after the first immunization (13%, day 2), moreover, differences in body weight when compared to controls were transient. This might indicate that administration of α-PA vaccine through IN route induces a mild to moderate dose-dependent toxic effect. When using IN doses of the live α-PA vaccine lower than 3×10^7^ CFU (8×10^5^ CFU and 7×10^6^ CFU, *n* = 8 mice/group), vaccinated mice presented similar outcomes as controls after challenge with PA14, thus suggesting no protective effect (S17 Fig).

We also tested a combination of IN plus IM immunization with 3×10^7^ CFU of α-PA vaccine (S16D Fig): one IN plus IM administration on days 0 and 14, and extra IM immunization on day 7. Here, we observed some weight loss (not significant) after the first and second vaccine administrations, concurring with IN immunizations, but not after IM administration, on day 7. This might support that combining these two immunization routes does not deepen vaccine toxicity. Again, differences in body weight in vaccinated mice were transient when compared to controls.

Further, we evaluated if the α-PA vaccine could colonize and multiply in the host, after IN or IM administration. Thus, we measured α-PA vaccine loads in the blood, lungs and livers of IN- (*n* = 4) and IM- (*n* = 4) vaccinated mice (3×10^7^ CFU). No CFUs were detected in the blood of mice as soon as 2 h after vaccination. Also, lungs and livers obtained on day 8 were sterile. Thus, it seems unlikely that α-PA vaccine strain can colonize the host.

### Combination of IN plus IM immunization with α-PA vaccine confers long-term protection against pneumonia

**Fig 5:**
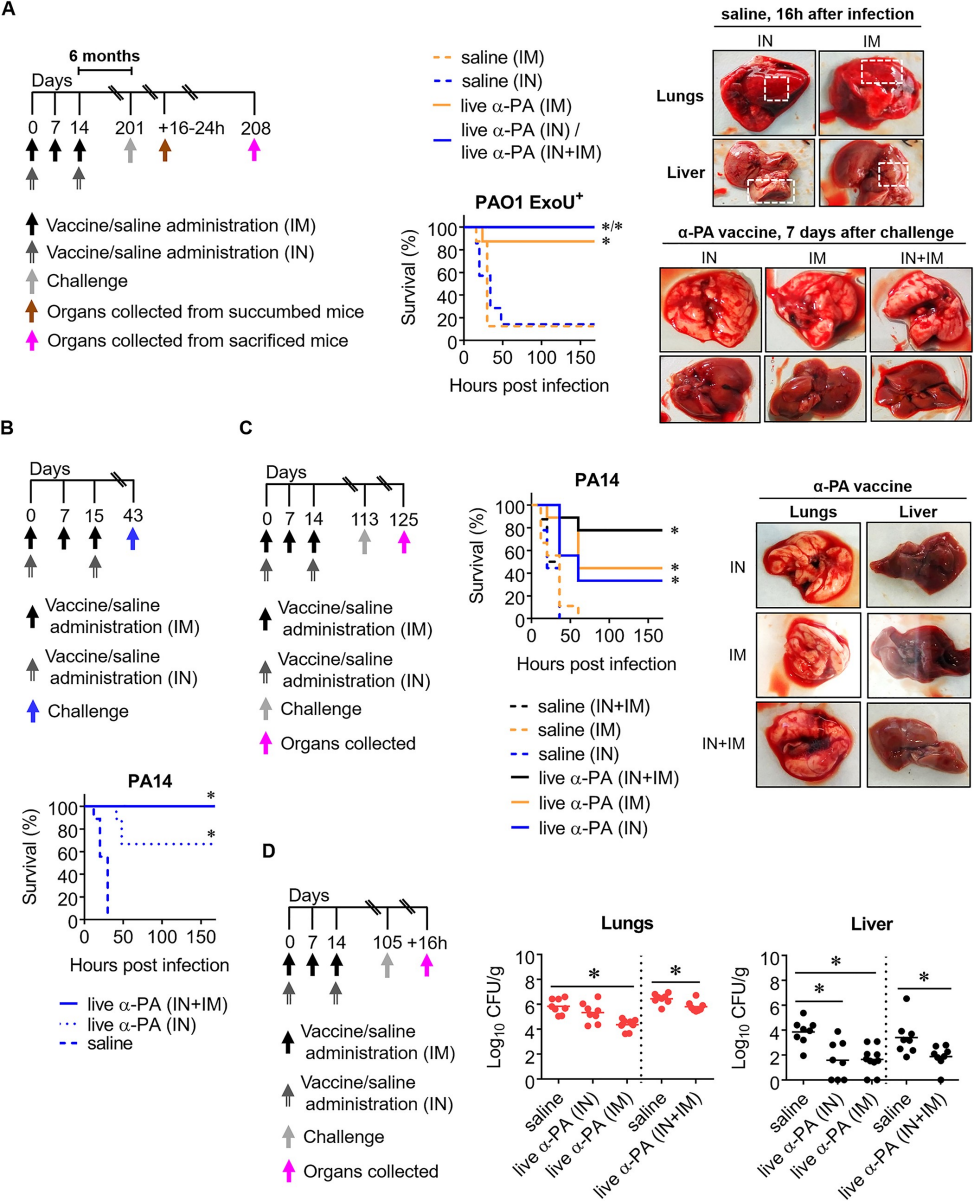
Combination of IN plus IM immunization with α-PA vaccine and protection against pneumonia. **(A)** Immunization schedule and mice survival after IN plus IM immunization with α-PA vaccine (3×10^7^ CFU) (IN+IM, *n* = 7), IM immunization with α-PA vaccine (3×10^7^ CFU, *n* = 8), IN immunization with α-PA vaccine (3×10^7^ CFU) (IN, *n* = 5), or saline administration (IM, *n* = 8; IN, *n* = 8) and challenge with PAO1 ExoU^+^ (4×10^5^ CFU). *P<0.05 (log-rank test). Images show lungs and livers collected from saline mice which succumbed to infection 16 h after challenge and from random vaccinated mice, sacrificed one week after challenge. Dashed squares indicate pulmonary hemorrhages and pale zones in livers. **(B)** Immunization schedule and mice survival after IN plus IM immunization with α-PA vaccine (3×10^7^ CFU) (IN+IM, *n* = 9), IN immunization with α-PA vaccine (3×10^7^ CFU) (IN, *n* = 9), or saline administration (IN+IM, *n* = 9) and challenge with PA14 (1×10^6^ CFU). *P<0.05 (log-rank test). **(C)** Immunization schedule and mice survival after IN plus IM immunization with α-PA vaccine (3×10^7^ CFU) (IN+IM, *n* = 9), IM immunization with α-PA vaccine (3×10^7^ CFU, *n* = 9), IN immunization with α-PA vaccine (3×10^7^ CFU) (IN, *n* = 9), or saline administration (IN+IM, *n* = 8; IM, *n* = 9; IN, *n* = 9) and challenge with PA14 (1×10^6^ CFU). *P<0.05 (log-rank test). Images show lungs and livers collected 12 days after challenge from random vaccinated mice. **(D)** Immunization schedule and bacterial loads in lungs and livers of mice after IN plus IM immunization with α-PA vaccine (3×10^7^ CFU) (IN+IM, *n* = 8), IM immunization with α-PA vaccine (3×10^7^ CFU, *n* = 10), IN immunization with α-PA vaccine (3×10^7^ CFU) (IN, *n* = 8), or saline administration (IN+IM, *n* = 8; IN, *n* = 8) and challenge with PA14 (1×10^6^ CFU). *P<0.05 (Kruskal-Wallis and Mann-Whitney U test were used for 3- and 2-group comparison, respectively).

Body weight monitoring showed that using a two-dose regimen with 3×10^7^ CFU of α-PA vaccine administered IN can be less toxic than using 2×10^8^ CFU. To test the efficacy of this alternative dosing regimen, we challenged mice with PAO1 ExoU^+^ using the previous model of lethal lung infection. We simultaneously tested protection in the long term, challenging mice 6 months after the last immunization (Fig 5A). As shown, survival of vaccinated mice (IN route) was 100%, whereas 86% of control mice died. Thus, IN immunization using 3×10^7^ CFU of α-PA vaccine offered considerable protection in the long-term. Thus, we used this dose thereafter. When the vaccine was administered IM (using 3×10^7^ CFU) or combining IN plus IM routes, 88% and 100% mice survival was observed, respectively, being in line with the previous findings suggesting that IM route is not as protective as the IN one. In this experiment, we also performed a macroscopic observation of lungs and livers collected from mice at two different time points. The first analyzed group were those mice which succumbed to infection between 16 and 24 h after challenge. On this occasion, all mouse lungs showed a visible and disseminated bloody appearance, indicating severe congestion and intra-alveolar hemorrhage. In turn, livers were paler than normal, suggesting inflammation. The second group were surviving mice, which were sacrificed 7 days after challenge. Lungs from these animals had no noticeable signs of haemorrhages and livers had no visible signs of inflammation.

Then, we compared the protective effect of α-PA vaccine using the IN, and the combination of IN plus IM routes against PA14 lethal pneumonia. As shown in Fig 5B, IN-vaccinated mice showed only 67% survival, whereas 100% of mice administered the second regimen survived. This suggests that IN vaccination alone is not as effective against PA14 as the IN plus IM combination. Next, we performed the same experiment, but using also the IM route and challenging mice on day 113 (~3 months after the last immunization) (Fig 5C). A substantial decrease on survival was observed for IN and IM routes of administration; 33 and 44%, respectively. Still, IN plus IM administration of α-PA vaccine resulted in 78% survival. Lungs and livers randomly collected from vaccinated mice which survived to challenge neither presented PA14 bacterial counts nor visible pathology (Fig 5C, right panel).

Further, we measured PA14 bacterial loads in the lungs and livers of vaccinated and control mice challenged ~3 months after the last immunization, in order to find a correlation between bacterial burden and the survival observed in the previous experiment (Fig 5C). As shown in Fig 5D, high CFU counts were obtained in the lungs of all mice, but with slight reduction in vaccinated mice (IM and IN+IM). Vaccination resulted in all cases in a more pronounced reduction in liver bacterial loads, with undetected levels in some vaccinees. No clear correlation could be established between CFU and protection observed in the long-term with the different immunization routes, however, it seems likely that vaccination reduces the local and systemic bacterial burden.

### Histological analysis of lungs after α-PA vaccination and PA14 infection

**Fig 6:**
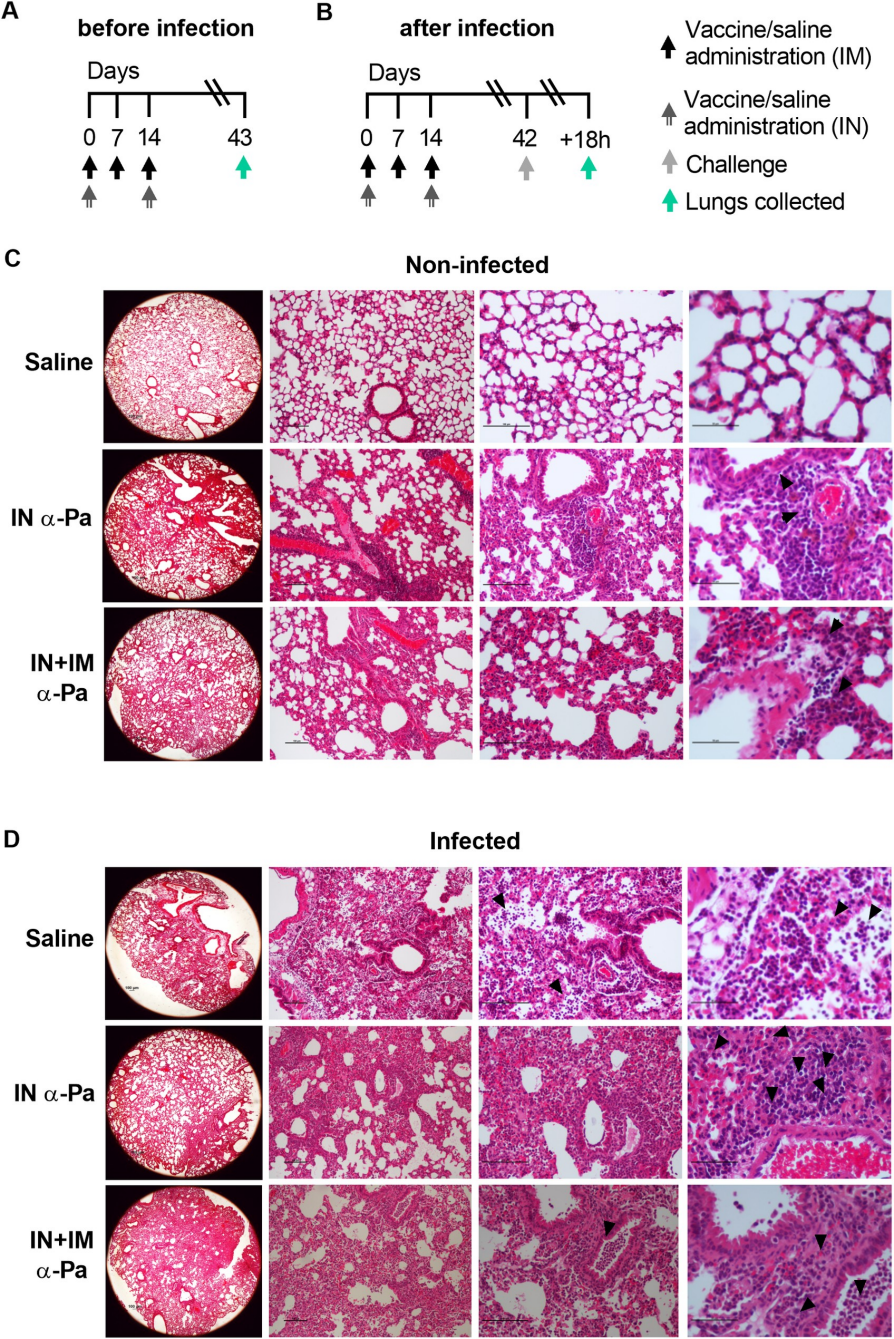
Lung histopathology caused by PA-pneumonia in vaccinated and naive mice. **(A)** Immunization and lung collection schedule for **(C). (B)** Immunization, challenge and lung collection schedule for **(D). (C)** Representative photomicrographs of histological sections of lungs obtained from mice after IN (IN) immunization with α-PA vaccine (3×10^7^ CFU) (*n* = 3), IN plus IM (IN+IM) immunization with α-PA vaccine (3×10^7^ CFU) (*n* = 3) and saline administration (IN+IM) (*n* = 3). **(C)** Arrows show perivascular and periarteriolar foci of inflammatory infiltrate of the mononuclear type in the parenchyma. **(D)** Representative photomicrographs of histological sections of lungs obtained from mice after IN (IN) immunization with α-PA vaccine (3×10^7^ CFU) (*n* = 6), IN plus IM (IN+IM) immunization with α-PA vaccine (3×10^7^ CFU) (*n* = 5), saline administration (IN+IM) (*n* = 5) and challenge with PA14 (1×10^6^ CFU). **(D)** Arrows show a severe inflammatory infiltrate of the polymorphonuclear type dispersed throughout the pulmonary parenchyma. Arrowheads show polymorphonuclear infiltrate filling most of the alveoli (saline), areas of scarce inflammatory mononuclear infiltrate (IN-immunized mice) and areas of inflammatory infiltrate of the polymorphonuclear type leukocyte with intrabronchial location (IN+IM-immunized mice), respectively. **(C, D)** The columns show images with ×25 (left), ×100 (center left), ×200 (center right) and ×400 (right) magnifications.

We assessed the differential outcomes of non-infected and infected mice after immunization (IN and IN plus IM administration) and control mice, by histological analysis of lungs (Fig 6). As shown in Fig 6C, the lungs of mice given α-PA vaccine showed some perivascular and periarteriolar foci of inflammatory infiltrate of the mononuclear type (arrows), evidencing mild to moderate inflammation, but with overall preservation of alveolar and airway architecture. Non-infected control mice showed no signals of inflammation. Lung histopathology of vaccinated and non-vaccinated mice subsequently infected with PA14 (Fig 6D) showed extensive polymorphonuclear (PMN) infiltration dispersed throughout the pulmonary parenchyma (arrows). This is consistent with recruitment of inflammatory cells and the role of neutrophils as essential mediators of host defense in the lungs against PA infection[27]. However, whereas sham-immunized mice presented PMN infiltrates filling most of the alveoli (arrowheads), vaccinated mice showed scarce alveolar infiltration, which might indicate less severe disease due to the reduced presence of tissue-injuring neutrophils[28] in these areas (alveoli preservation).

Overall, these results suggest that nasal vaccination with α-PA vaccine protects animals from the extensive bacterial dissemination in the lungs triggered by infection, thus it might reduce the damaging effects caused by neutrophil invasion of alveoli.

### Mucosal antibody responses after immunization with α-PA vaccine

**Fig 7:**
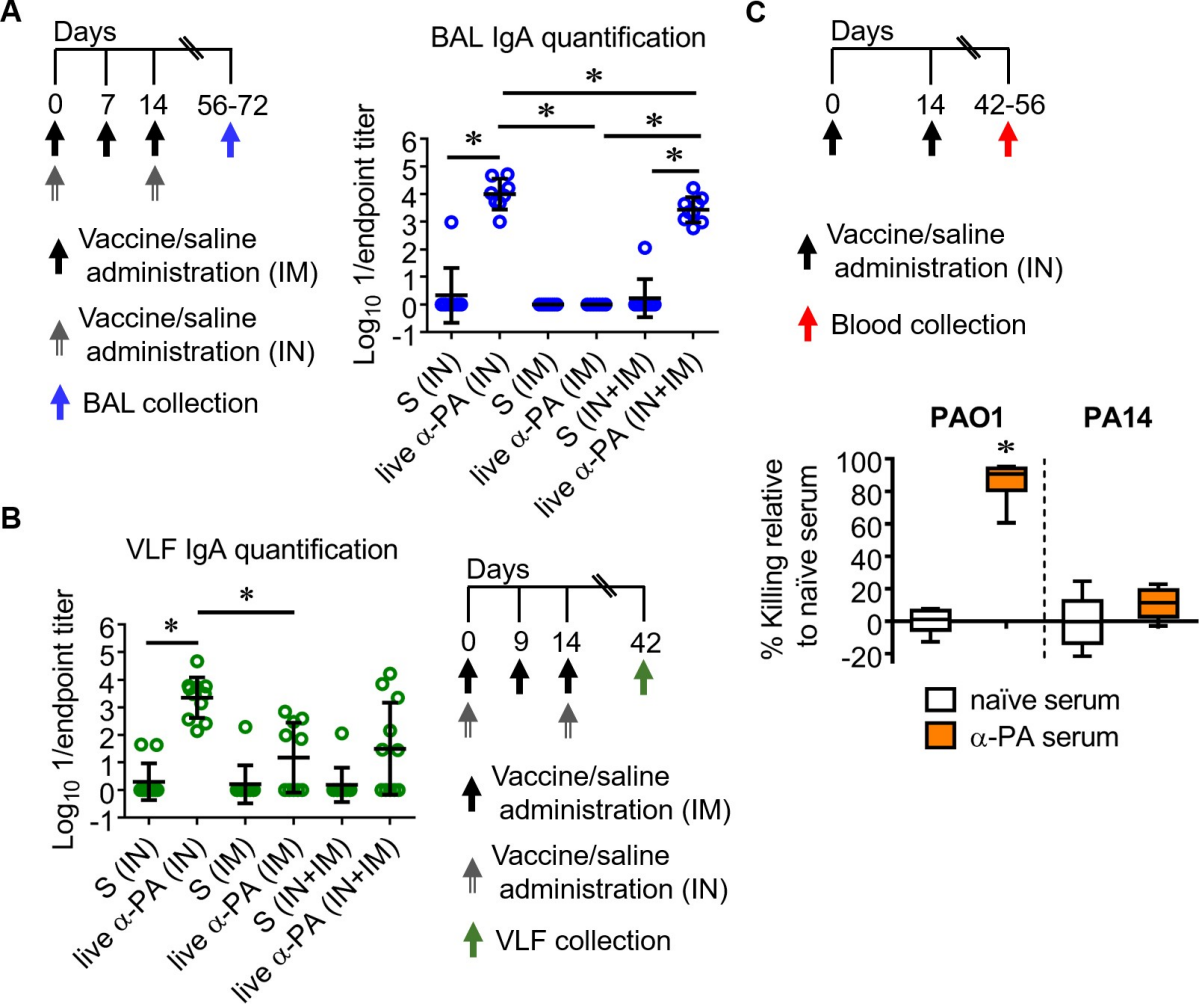
Live α-PA vaccine generates mucosal antibody responses and functional IgG in blood. **(A)** Immunization schedule and IgA titers in BAL after IN plus IM immunization with α-PA vaccine (3×10^7^ CFU; IN+IM, *n* = 9), IM immunization with α-PA vaccine (3×10^7^ CFU; IM, *n* = 7), IN immunization with α-PA vaccine (3×10^7^ CFU; IN, *n* = 8), or saline administration (IN+IM, *n* = 9; IM, *n* = 9; IN, *n* = 9). Titers were normalized respective to 50 μL. **(B)** Immunization schedule and IgA titers in VLF after IN plus IM immunization with α-PA vaccine (3×10^7^ CFU; IN+IM, *n* = 8), IM immunization with α-PA vaccine (3×10^7^ CFU; IM, *n* = 7), IN immunization with α-PA vaccine (3×10^7^ CFU; IN, *n* = 8), or saline administration (IN+IM, *n* = 8; IM, *n* = 8; IN, *n* = 8). **(A, B)** S, saline. *P<0.05 (Kruskal-Wallis test), between the indicated groups. **(C)** Immunization schedule and *in vitro* OPKA assay, representing percent killing of PAO1 (2×10^4^ CFU) and PA14 (2×10^4^ CFU) by α-PA serum (or naïve serum) in the presence of human PMNs. α-PA and naïve sera were obtained from the blood of mice after IN immunization with α-PA vaccine (3×10^7^−2×10^8^ CFU, *n* = 6) or saline administration (*n* = 6), respectively. *P<0.05 (Mann-Whitney U test).

We investigated the local and distal mucosal responses to α-PA vaccine after immunization of mice via IN, IN plus IM and IM routes. IgA antibodies were quantified in bronchoalveolar lavages (BAL) and vaginal lavage fluids (VLF) (Fig 7A and 7B). Significantly increased IgA levels were detected in the BAL of IN- and IN plus IM-vaccinated mice, with the higher amounts being produced after IN immunization. IN immunization stimulated IgA production also in VLF. IM immunization elicited a slight increase in VLF IgA antibodies. Therefore, we hypothesize that intranasal vaccination might influence mucosal immunity via IgA secretion.

### IN immunization with α-PA vaccine induces functional antibodies against LPS-homologous serogroup

We previously observed that α-PA-IN-vaccinated mice challenged with a PAO1 sepsis presented 88.9% survival, whereas all control mice died[11]. Based on this precedent, we hypothesized that antimicrobial activities of serum antibodies generated by IN immunization might also present complement-mediated killing and opsonophagocytosis against PA. Thus, we measured PAO1 and PA14-specific opsonophagocytic killing antibody (OPKA) activities, using α-PA serum obtained from the blood of mice after IN immunization and naïve serum from control mice. As illustrated in Fig 7C, there was significant killing of PAO1 in the presence of α-PA serum and human PMNs, but not of PA14. For PAO1, no significant killing was observed without PMNs (0 *vs* 19% killing; naïve *vs* α-PA serum, respectively). These data are consistent with the previous observed lack of passively-transferred protection (S15 Fig), and suggests that an antibody-independent mechanism might be responsible for the α-PA vaccine-mediated protection against the LPS-heterologous strain PA14.

### Exploring alternative routes of immunization for mucosal immunity

We also tested the protective mucosal immunity generated by intradermal (ID) and subcutaneous (SC) delivery, considering that these immunization routes evoke both systemic and mucosal immunity. No body weight loss was observed for mice vaccinated using ID and SC routes (S18A and S18C Fig), resembling the previous results obtained with IM immunization. However, 6 days after the first ID injection of α-PA vaccine (1.5×10^8^ CFU), all mice presented skin lesions at the inoculation site (S18B Fig). These lesions were transient, not being appreciable beyond day 9. Likewise, 2 out of 8 mice developed lesions in the neck area 6 days after SC immunization (1.5×10^8^ CFU) (S18D Fig). Again, these lesions remitted 3 days after. These results suggest that α-PA vaccine might elicit transient local adverse reactions when injected ID and SC at high doses as 1.5×10^8^ CFU.

Then, mice were challenged IN with PA14 (S19 Fig). As shown in S19B Fig, all mice ID-administered 1.5×10^8^ CFU of α-PA vaccine survived infection, whereas all control mice succumbed to acute pneumonia. Mice administered 3×10^7^ CFU of α-PA vaccine presented 67% survival. Thus, it seems likely that α-PA vaccine administered ID confers a significant protection against acute lung infection caused by PA14, being apparently more effective when using higher doses as 1.5×10^8^ CFU. Increased IgG levels were observed in all immunized mice (S19C Fig). IgG titers of 3×10^7^ CFU-vaccinated mice were not lower than when 1.5×10^8^ CFU were used.

Then, we tested vaccine efficacy using SC route. As illustrated in S7E Fig, mice administered 1.5×10^8^ CFU and 3×10^7^ CFU of α-PA vaccine presented 88 and 25% survival, respectively, suggesting that SC route might not elicit the same level of protection as the ID route. Again, increased IgG levels were present in all immunized mice (S19F Fig), but no differences were observed between 3×10^7^ CFU and 1.5×10^8^ CFU-vaccinated mice. This observation is consistent with the results obtained when using ID immunization, supporting that mucosal protection observed may not depend on systemic antibodies alone.

## Discussion

PA is among the several highly resistant gram-negative pathogens identified as emerging pathogens in both the US and other parts of the world, a group named ESKAPE[29]. It is also a leading pathogen causative of nosocomial infections, frequently originating acute pneumonia associated with mechanical ventilation (VAP)[30]. PA-derived VAP has a high attributable mortality, ranging from 13.5%[31] to 38%[32]. The worldwide increasing prevalence of MDR and XDR-resistant PA is of grave concern, making it imperative to develop a vaccine to protect against pneumonia. In our previous study, a D-Glu auxotrophic strain was selected and tested as a vaccine prototype against PA-derived sepsis using intraperitoneal immunization[11]. Nevertheless, injection of substances into the peritoneal cavity is rarely used in larger mammals and humans. Now, this work aims at identifying whether this D-Glu auxotroph holds the potential to be immunogenic and effective in a model of lethal pneumonia. Although other routes were tested (IM, SC and ID), we focused on IN immunization, as nasal mucosa provides a universal entry portal for PA, inducing local mucosal immune responses[33]. When administered by this route, our live vaccine elicited both humoral and cellular immunity and reduced the number of non-surviving mice considerably after infection with two ExoU-positive PA strains. Some protection against the ST235 XDR disseminated clone was also observed. Although ExoU-positive strains are not responsible for the majority of hospital-acquired PA infections, we selected these for challenge as mice are more likely to die than those inoculated with ExoU-negative strains. Moreover, ExoU is the most potent cytotoxin, present in approximately 30% of strains associated with the most severe infections, including septicemia [34,35]. Thus, the relative virulence associated with this type III effector protein may have more important prognostic implications for patients infected with PA.

Despite protection levels for PAO1 ExoU^+^ and PA14 were below 100% in some occasions, a trend toward reducing disease severity was observed when using IN immunization. However, none of the immunological determinants investigated could explain the unequal protection against ST235. According to our observation that IN immunization using a lower vaccine dose (3×10^7^ CFU) resulted in 67% survival of mice infected with PA14 in contrast to the 88% observed when using 2×10^8^ CFU, it might be hypothesized that using higher vaccine doses can compensate for the low vaccine efficacy observed against ST235 strain; however, we could not test this hypothesis due to the safety margin for IN administration.

Numerous studies have shown a host protective role of IL-17A in PA pneumonia[21,36,37]. IL-17-producing γδ T cells have been shown to promote neutrophil chemotaxis, eliminating PA bacteria during acute pulmonary infection[38] and to be involved in B cell activation[39]. However, our results appear to indicate that CD4^+^ T helper cells, rather than γδ T cells are stimulated by the α-PA vaccine to produce IL-17A as the IN-immunized mice presented increased frequencies of IL-17A^+^CD4^+^ cells in lungs than controls, while IL-17-producing γδ T cells were found at similar frequencies in both groups. IL-17A produced by Th17 cells is therefore likely implicated in the protective effect of the α-PA vaccine against acute lung infection. Indeed, this T cell population has been previously implicated in vaccination-induced protection against PA[21,36] and other bacteria causative of pulmonary infections[40].

The protection conferred by IL-17A has been associated with a rapid recruitment of neutrophils to the airways and the subsequent reduction of bacterial load[41]. Accordingly, vaccinated mice presented higher numbers of neutrophils in the lungs than controls. Despite having a marked neutrophil infiltration in the pulmonary parenchyma, vaccinated mice nevertheless presented markedly lower transmigration of neutrophils into the alveolar airspace, which is associated with damage to alveolar epithelium and impaired alveolar function[28]. Mononuclear CD11b^+^ cells, present at elevated numbers in the lungs of vaccinated mice might contribute to the lower inflammatory pathology in these mice as it has been previously shown in a condition of excessive lung inflammation[42]. Macrophages, which were found more abundant in the lungs of vaccinated mice might also contribute for protection against excessive inflammation. Indeed, host tissue-damaging inflammation in mice infected with PA was associated with lower macrophage bactericidal activity[43]. The mononuclear cell response that was observed in vaccinated mice, but not in controls, might thus explain the higher host protection at lower inflammatory cost.

Serum obtained from IN-vaccinated mice showed opsonic killing activity against PAO1. Thus, serum antibodies raised by immunization might thus mediate antimicrobial functions through opsonization, either by promoting complement deposition and cell lysis, and/or by promoting phagocytosis and killing by innate immune cells via Fc receptor recognition. This could help reduce or prevent bacterial dissemination into the circulation, acting synergistically with IgA in the lungs to prevent disease exacerbation. However, the absence of opsonic killing activity against PA14 suggests that an antibody-independent mechanism as T-cell help could be necessary to promote effective killing of the PA14 strain by phagocytic cells.

IM immunization with α-PA vaccine evoked titers of serum antibody comparable to the ones detected upon IN immunization. However, reduced protection against PA14 strain was observed. Taken together the results from passive immunization experiments, OPKA, flow cytometry and cytokine quantification, it is likely that the lower protection observed after IM immunization is associated with lower activation of cellular immunity (including lower Th17 activation). This hypothesis is further supported by recent findings describing an association between local IgA responses and Th17 establishment in the lungs after airway immunization [44]. No IgA was detected in BAL of IM-immunized mice, accordingly. Indeed, increased susceptibility to PA-pneumonia was observed in patients with secondary IgA deficiency[45].

Despite IN administration of 2×10^8^ CFU of α-PA vaccine offered better improvement in mice survival than IM route, it nevertheless induced significant body weight loss and mild inflammation in the lungs. This may be due to intrinsic pathogenicity of the PA strain PAO1[46], emphasizing the need for using low doses. Indeed, when using a lower dose of α-PA vaccine (3×10^7^ CFU), body weight losses were transient and no more present one week after each immunization. This effect was considered of mild to moderate toxicological relevance, and appeared to be dose-dependent. Thus, the IN immunization route using 3×10^7^ CFU was combined with IM delivery, the last one conferring reduced protection but no significant body weight loss. The combined IN plus IM administrations with α-PA vaccine was apparently more effective in reducing disease severity that IN vaccination alone, as survival of mice was superior. Therefore, combining the two immunization routes could be an option to overcome the toxic effect of IN administration.

We also tested the protective effect generated by ID and SC delivery as these vaccination routes were reported to induce both systemic and mucosal immune responses[47,48]. ID and SC administration of α-PA vaccine offered significant protection against PA14 when using high vaccine doses, however, transient local adverse reactions were observed and this side-effect should be considered in the future.

The results obtained in this study are still limited due to the novelty of this D-Glu auxotrophic vaccine, impeding an accurate comparison with other reported α-PA vaccines. Indeed, the most similar vaccine candidates described are live-attenuated *aroA* deletants of PAO1[49] and PA14[21], but none of these strategies rely on using bacterial auxotrophy for D-Glu. Perhaps, some similarities can be established with BPZE1, a genetically-modified *Bordetella pertussis* strain which was developed as a live attenuated nasal pertussis vaccine by genetically eliminating or detoxifying 3 toxins[50]. BPZE was shown to be safe in humans[51], able to transiently colonize the human nasopharynx and to induce antibody responses, when using 10^3^, 10^5^ and 10^7^ CFU through IN delivery. However, despite dose optimization regarding α-PA vaccine should be considered in future studies, it may be reasonable to hypothesize that this vaccine may similarly provide an acceptable level of efficacy without compromising safety. Moreover, refinement of this prototype vaccine through deletion of virulence genes could be explored to abrogate the toxic effect observed. An additional limitation is that *in vivo* studies were performed in BALB/c mice only, and differences in immunity may exist if other mouse strains are used.

While several candidates to receive α-PA vaccination have been discussed[8], those patients scheduled for cardiac surgery[52] and those undergoing mechanical ventilation[53] appear to be the most obvious target individuals. Another important infection group are those undergoing chemotherapy[54] or AIDS patients[55]. But because live vaccines are generally contraindicated for patients with severe immunocompromised status, some debate exists about targeting these individuals. However, as live attenuated Salmonella expressing PA-O antigen was immunogenic and effective in immunocompromised mice [56], it seems reasonable to consider the use of auxotrophic live vaccines in the future, after further virulence attenuation.

In summary, these findings suggest that the live α-PA vaccine described herein could be a promising approach to achieve mucosal immunity against pneumonia caused by highly pathogenic PA strains and possibly those that are refractory to broad-spectrum antibiotics. Immunization using the IN route seems favorable in comparison to conventional parenteral delivery routes, enabling local as well as systemic immunity and conferring an improvement in survival in a model of lethal pneumonia. However, further studies on the effects of eliminating toxicity genes on the PAO1 vaccine-strain background are needed to consider nasal administration.

## Materials and methods

### Ethics Statement

All animal experiments were done with the approval of *Comité Ético de Experimentación Animal of Complejo Hospitalario Universitario A Coruña*, *Spain*, under project license number 15002/2016/08 and in compliance with the *Real Decreto 53/2013*, *of 1*^*st*^ *february*, which establishes the basic rules applicable for the protection of animals used in experimentation and other scientific purposes, including teaching.

### Bacterial strains and culture conditions

All bacterial strains and plasmids used in this study are listed in S1 Table. All PA strains were grown in Luria-Bertani broth (LB) or on LB agar at 37°C. When indicated, D-Glu was used at 10 mM.

### Animals

All mice were maintained in the specific pathogen-free facility at the Centro Tecnológico de Formación de la Xerencia de Xestión Integrada A Coruña. Male and female BALB/c mice were bred in our colony and used for first time procedures between the ages of 6 and 8 weeks. Studies were not blinded.

### Immunization and lethal pneumonia challenge

To prepare inocula for active immunizations and infections, bacteria were cultured at 37°C under agitation (180–210 rpm) until reaching OD_600nm_ = 0.7. Cultures were harvested by centrifugation (4,000 g, 20 min, 4°C), pellets washed twice, suspended and adjusted in sterile NaCl 0.9% to different doses (vaccine vehicle was always NaCl 0.9%—saline). Prior to mice inoculation, bacterial inocula were quantified by CFU enumeration in agar plates. In detail, PA and PA Δ*murI* (α-PA vaccine) strains were cultured in LB and LB with D-Glu, respectively, at 37°C and 180 rpm, harvested by centrifugation, washed twice with LB and finally adjusted with saline as above. To prepare formalin-inactivated PAO1, cultures were harvested by centrifugation, washed with saline and inactivated in 1% (w/v) paraformaldehyde in PBS by incubation at 37°C under agitation (180 rpm) for 2 h. The bacteria were then washed twice with NaCl 0.9% and adjusted as before. Killing of bacteria was checked by plating on LB agar to determine the absence of colonies after overnight incubation. For active immunization assays, mice were inoculated IN with α-PA vaccine (or formalin-inactivated PAO1) with a total volume of 20 μL. Control mice were administrated saline. For IM, SC and ID injections of α-PA vaccine, we used a total volume of 100 (half volume per leg), 200 and 40 μL, respectively. Four weeks after the last immunization (at least), mice were anesthetized and IN-challenged (20 μL) with virulent PA strains. To facilitate migration of the inoculum to the alveoli, mice were held in a head-up vertical position for 2 min. Then, mice were monitored for mortality.

### Sampling of mice

Blood samples were collected from the submandibular vein of anesthetized mice, and sera were separated from the blood cells by centrifugation (1,500 g, 15 min). To assess bacterial burden in lungs and livers, mice were euthanized at indicated time points, tissues were extracted aseptically, homogenized in sterile NaCl 0.9% and CFU enumerated by plating 10^−2^-fold serial dilutions in agar plates. VLF was obtained by washing the vagina of female mice twice with 50 μL sterile NaCl 0.9%, then VLF was centrifuged and the clear supernatant was used for the assays. BAL was performed by insertion of a catheter in the trachea of terminally anesthetized mice, through which 5–8 mL saline sterile solution was instilled into the bronchioles and gently retracted to maximize fluid retrieval.

### ELISA

Quantification of IgM, IgG, IgG1, IgG2a, IgG2b and IgG3 in mouse sera was performed using an indirect ELISA as described in[11]. Hk PAO1 and PA14 were obtained by growing cells in LB at 37°C under agitation (180 rpm) until reaching OD_600nm_ = 1.0. Then, bacteria were harvested by centrifugation (3,900 g, 20 min, 4°C), washed, suspended in sterile NaCl 0.9% and heat-inactivated by incubation at 100°C for 2 h. Killing of bacteria was checked by plating on LB agar and overnight incubation at 37°C. Quantification of IgA antibodies in VLF and BAL was performed by indirect ELISA according to[57], using secondary antibody alkaline phosphatase-conjugated anti-mouse IgA diluted 1:500. Plates were “coated” with formalin-inactivated PAO1, which was fixed to the bottom of the wells after overnight incubation at 4°C in 100 mM carbonate-bicarbonate buffer, pH 9.6. IgA titers in BAL were normalized respective to 50 μL, the same volume as those tested for VLF samples.

### Cytokine detection in splenocyte cultures

Spleen single cell suspensions suspended in RPMI-1640 complete medium were plated in round-bottom 96-well plates (5×10^5^/well) and stimulated with Hk PA14 (5×10^5^ CFU and 5×10^4^ CFU) at splenocytes:bacteria ratios of 1:1 and 1:0.1 for 3 days at 37°C and 5% CO_2_. Hk PA14 was obtained as described above, but growing cells in LB until reaching OD_600nm_ = 0.7. Then, bacteria were heat-inactivated as before. Control splenocytes were added RPMI-1640 only (no stimuli). Samples from all animals were used in duplicate wells. The concentrations of IFN-γ, IL-17A, and IL-4 in cell culture supernatants were quantified using Ready-SET-Go! Mouse IFN-γ, IL-17A and IL-4 ELISA kits (eBioscience), respectively. IL-10 concentration was determined using the Mouse IL-10 DuoSet ELISA development system (R&D Systems).

### Flow cytometry analysis

The assessment of cell surface lineage or activation markers on different splenic and lung leukocyte populations was performed by flow cytometry analysis (fluorescence-activated cell sorter [FACS] analysis), as described in Supporting Information, S1 Material and methods.

### Passive immunization

Mice were given IgG-enriched antiserum (100 μL IP, 400 μg IgG/mouse), or naïve serum, 4 hours prior to the challenge with PA14. IgG-enriched antiserum was prepared using purified antibodies obtained from IN- and IM-immunized mice using the previous vaccination regimens (Figs 1A and 4A), which were then diluted in naïve serum. Antibody purification was carried out using the Protein G GraviTrap gravity-flow columns (GE Healthcare Life Science, Inc., USA). Amicon Ultra-15 filter devices (Millipore, Inc., USA) were used thereafter for desalting and concentration of eluted antibodies.

### Histopathology

Lungs were harvested and fixed in 4% paraformaldehyde pH 7.0 for a minimum of 24h. Fixed tissues were then routinely processed and embedded in paraffin, cut in 3 μm sections and stained with hematoxylin and eosin (HE) after dewaxing in xylene and rehydration in decreasing ethanol concentrations, for histologic evaluation by a pathologist. The lung sections throughout the entire organ were microscopically evaluated to assess the distribution and character of pathologic alterations, as well as the lung inflammation pattern. All examinations were performed by a trained veterinary experimental pathologist blinded to the experimental conditions.

### Opsonophagocytic killing assay (OPKA)

OPKA was assessed against PAO1 as previously[11], using α-PA serum obtained from the blood of mice after IN immunization and naïve serum from control mice. The target strains, 5×10^5^ polymorphonuclear leukocytes (PMNs) from human volunteers, 2.5% complement serum from rabbit and test sera (1:8 final dilution) were all prepared in RPMI 1640 medium containing 10% FBS. PMNs were obtained from whole blood using a single Percoll density gradient[58].

### Statistics

Data presented in box plots were analyzed using Mann Whitney test for 2 experimental groups-comparison and Kruskal-Wallis for 3 or more groups-comparison (with Dunn’s multiple comparison test). Otherwise, means were compared by using Student’s t test. Survival data were compared using the log-rank test. Statistical significance for mice weight loss was assessed by mixed ANOVA with “study day” as within-subjects factor and “treatment group” as between-subjects factor, followed by Bonferroni’s Multiple Comparison Test. Sphericity was checked via Mauchly’s test. Differences were considered significant when *P*<0.05. Significant changes within groups due to mice body weight gain along the time are not shown for clarity.

## Supporting information

S1 Protocol Flow cytometric analysis. (DOCX)

S1 Fig Gating strategy used to define CD4 T naïve and antigen-experienced cells in the spleens of non-infected mice. Debris were excluded (Cell minus debris) based on FSC and SSC parameters and doublets were excluded (Single cells) in FSC-H versus FSC-A plots. Viable cells (Live cells) were gated as fixable viability dye negative cells. CD4 T cells were defined as CD3^+^CD4^+^ and quadrants were set, based on FMO stainings on contour plots, to define naïve (CD62L^+^CD44^-^), central memory (CD62L^+^CD44^+^) and effector memory (CD62L^-^CD44^+^) cells.
(JPG)

S2 Fig Cytokine differentiation profile of CD4^+^ T cells from mice IN-administered α-PA vaccine, before and after infection with PA. **(A)** BALB/c mice (*n* = 6/group) were immunized with live α-PA vaccine (2×10^8^ CFU) or administered saline, according to the schedule; then spleens and lungs were collected on the day indicated. **(B)** BALB/c mice (*n* = 6/group) were immunized with live α-PA vaccine (2×10^8^ CFU) or administered saline, according to the schedule; then mice were infected with PA14 (1×10^6^ CFU) on the day indicated. Spleens and lungs were collected 9 hours after. **(C)** Frequency (percentage) of splenic and lung CD4-gated T cells expressing IFN-γ, IL-10, IL-4 and TNF-α of infected and non-infected mice, detected by intracellular staining after stimulation with PMA/ionomycin. Bars represent mean ± SD of data. *P<0.05, **P<0.01, ***P<0.001 (t-test), compared with saline group. *ns*, not significant.
(JPG)

S3 Fig Cytokine differentiation profile of splenic CD4^+^ T cells from mice IN-administered α-PA vaccine, before and after infection with PA, detected by intracytoplasmic staining and flow cytometric analysis. **(A)** BALB/c mice (*n* = 6/group) were immunized with live α-PA vaccine (2×10^8^ CFU) or administered saline, according to the schedule; then spleens were collected on the day indicated. **(B)** BALB/c mice (*n* = 6/group) were immunized with live α-PA vaccine (2×10^8^ CFU) or administered saline, according to the schedule; then mice were infected with PA14 (1×10^6^ CFU) on the day indicated. Spleens were collected 9 hours after. **(C)** Representative examples of CD4-gated T cells of spleens obtained from infected and non-infected mice, detected by intracellular staining, after stimulation with PMA/ionomycin. Analysis regions were set according to FMO and isotype control-stained samples. Numbers inside dot plot regions represent means ± SD of the frequency of cells due to respective cytokine staining.
(JPG)

S4 Fig Cytokine differentiation profile of CD4^+^ T cells from lungs of mice IN-administered α-PA vaccine, before and after infection with PA, detected by flow cytometric analysis. **(A)** BALB/c mice (*n* = 6/group) were immunized with live α-PA vaccine (2×10^8^ CFU) or administered saline, according to the schedule; then lungs were collected on the day indicated. **(B)** BALB/c mice (*n* = 6/group) were immunized with live α-PA vaccine (2×10^8^ CFU) or administered saline, according to the schedule; then mice were infected with PA14 (1×10^6^ CFU) on the day indicated. Lungs were collected 9 hours after. **(C)** Representative examples of CD4-gated T cells of lungs obtained from infected and non-infected mice, detected by intracellular staining, after stimulation with PMA/ionomycin. Analysis regions were set according to FMO and isotype control-stained samples. Numbers inside dot plot regions represent means ± SD of the frequency of cells due to respective cytokine staining.
(JPG)

S5 Fig Flow cytometry gating strategy used to define mouse spleen and lung CD4 T cells producing the cytokines IFN-γ, IL-17A, IL-10 and IL-4. Cells were stimulated with PMA/ionomycin in the presence of Brefeldin A for 4 h, followed by lineage marker surface staining. Produced cytokines were labelled by intracellular staining. Lymphocytes were selected based on FSC and SSC parameters, doublets were then excluded (Single cells) and viable cells (Live cells) were gated as fixable viability dye negative cells. CD4 T cells were defined as CD3^+^CD4^+^ and quadrants were set, based on FMO staining on contour plots, to define cytokine-producing cells. Representative contour plots of splenic CD4 T cells producing IFN-γ, IL-17A, IL-10, and IL-4, are shown.
(JPG)

S6 Fig Analysis of γδ T cells of mice IN-administered α-PA vaccine, before and after infection with PA. **(A)** BALB/c mice (*n* = 6/group) were immunized with live α-PA vaccine (2×10^8^ CFU) or administered saline, according to the schedule; then spleens and lungs were collected on the day indicated. **(B)** BALB/c mice (*n* = 6/group) were immunized with live α-PA vaccine (2×10^8^ CFU) or administered saline, according to the schedule; then mice were infected with PA14 (1×10^6^ CFU) on the day indicated. Spleens and lungs were collected 9 hours after. **(C, D)** Representative examples and frequency (percentage) of lung γδ-gated T cells expressing IL-17A **(C)** and IFN-γ **(D)** of infected and non-infected mice, detected by intracellular staining after stimulation for 4 h with PMA/ionomycin in the presence of Brefeldin A. Analysis regions were set according to FMO and isotype control-stained samples. Numbers inside dot plot regions represent means ± SD of the frequency of cells due to respective cytokine staining. Bars represent mean ± SD of data. *P<0.05, **P<0.01 (t-test), compared with saline group.
(JPG)

S7 Fig Gating strategy used to identify γδ T cells producing IFN-γ and IL-17A in mouse lungs by flow cytometric analysis. Cells were stimulated with PMA/ionomycin in the presence of Brefeldin A for 4 h, followed by lineage marker surface staining. Produced cytokines were labelled by intracellular staining. Debris were excluded (Cell minus debris), and lymphocytes were selected based on FSC and SSC parameters. Doublets were then excluded (Single cells), followed by dead cell exclusion (Live cells). γδ T cells were defined as γδ TCR^+^ and quadrants were set, based on FMO stainings to define cytokine-producing cells. Representative contour plots of lung γδ T cells producing IFN-γ and IL-17A are shown.
(JPG)

S8 Fig IN immunization with α-PA vaccine triggers IFN-γ, IL-17A and IL-10 cytokine secretion by mouse splenocytes. **(A, B)** Mice (*n =* 6-7/group) were immunized with α-PA vaccine (2×10^8^ CFU) or administered saline, according to the schedules; then spleens were collected. In **(B)**, mice were challenged with PA14 (1×10^6^ CFU), 9 h before the spleens were obtained. **(A, B)** Graphics show IFN-γ, IL-17A and IL-10 concentration in the supernatants of splenocyte cultures of vaccinated and non-vaccinated mice after being stimulated *ex vivo* with Hk PA14 (5×10^5^ CFU and 5×10^4^ CFU for 1:1 and 1:0.1 ratio of splenocytes:bacteria, respectively) for 3 days. No stimuli were added to control splenocytes. Mean ± SD. *P<0.05, (Mann-Whitney U test).
(JPG)

S9 Fig Analysis of myeloid cells in the lungs of IN α-PA vaccinated mice. **(A, B)** Mice (*n* = 6/group) were immunized with α-PA vaccine (2×10^8^ CFU) or administered saline, according to the schedule; then lungs were collected. In **(B),** mice (*n* = 6/group) were infected with PA14 (1×10^6^ CFU), 9 h before the lungs were obtained. **(C, D)** Total number of cells in the lungs of non-infected **(C)** and infected **(D)** mice, detected by flow cytometry upon surface staining, according to the schedules indicated in **(A)** and **(B)**, respectively. Analysis regions were set according to FMO control-stained samples. Bars represent mean ± SD of data. *P<0.05, **P<0.01, ***P<0.001, ****P<0.0001 (t-test).
(JPG)

S10 Fig Gating strategy used to identify the major myeloid cell subsets in mouse lungs by flow cytometric analysis. Cells isolated from collagenase-digested mouse lungs were stained with a mix of fluorophore conjugated antibodies after a pre-incubation step with anti-mouse CD16/CD32 (FcBlock). A sequential gating strategy was used to identify cell populations based on their surface expression of specific markers. After exclusion of debris (Cell minus debris) and doublets (Single cells), immune cells were gated based on CD45 expression (CD45^+^ cells), followed by dead cell exclusion (Live cells). Neutrophils were defined as CD11b^+^ Ly6G^+^ cells; dendritic cells (DC) were identified as Ly6G^-^ CD11c^+^ F4/80^-^ MHC class II^+^, that could be further divided into CD11b^+^ and CD11b^-^ DC subsets; alveolar macrophages (AM) were defined as Ly6G^-^ CD11c^+^ F4/80^+^ MHC class II^-^ and interstitial macrophages (IM) were set as Ly6G- CD11c^+^ F4/80^+^ MHC class II^+^; within the Ly6G^-^ CD11c^-^ CD11b^+^ F4/80^+^ population, cells were divided based on their Ly6C expression and side-scatter characteristics: Ly6C^+^ monocytes/macrophages (Ly6C^+^ Mo/MΦ) were defined as Ly6G^-^ CD11c^-^ CD11b^+^ F4/80^+^ Ly6C^+^, Ly6C^-^ Mo/MΦ as Ly6G^-^ CD11c^-^ CD11b^+^ F4/80^+^ and SSC^lo^ and eosinophils as Ly6G^-^ CD11c^-^ CD11b^+^ F4/80^+^ and SSC^hi^.
(JPG)

S11 Fig Memory CD4^+^ T cells response after IM immunization with live α-PA vaccine. **(A)** Mice (*n* = 6/group) were immunized with α-PA vaccine (3×10^7^ CFU) or administered saline, according to the schedule; then spleens were collected. **(B)** Percentage of spleen effector memory (CD44^+^ CD62L^-^), central memory (CD44^+^ CD62L^+^) and naïve (CD44^-^ CD62L^+^) CD4^+^ T cells. Mean ± SD of data. *P<0.05 (t-test).
(JPG)

S12 Fig Cytokine differentiation profile of CD4^+^ T cells from mice IM-administered α-PA vaccine, before and after infection with PA14. **(A)** BALB/c mice (*n* = 6/group) were immunized with live α-PA vaccine (3×10^7^ CFU) or administered saline, according to the schedule; then spleens and lungs were collected on the day indicated. **(B)** BALB/c mice (*n* = 6/group) were immunized with live α-PA vaccine (3×10^7^ CFU) or administered saline, according to the schedule; then mice were infected with PA14 (1×10^6^ CFU) on the day indicated. Spleens and lungs were collected 9 hours after. **(C)** Frequency (percentage) of splenic and lung CD4-gated T cells expressing IL-17A, IFN-γ, IL-10, IL-4 and TNF-α of infected and non-infected mice, detected by intracellular staining after stimulation with PMA/ionomycin. Bars represent mean ± SD of data. *P<0.05 (t-test).
(JPG)

S13 Fig Cytokine production after IM immunization with α-PA vaccine. **(A, B)** Mice (*n =* 6/group) were immunized with α-PA vaccine (3×10^7^ CFU) or administered saline, according to the schedules; then spleens were collected. In **(B)**, mice were challenged with PA14 (1×10^6^ CFU), 9 h before the spleens were obtained. **(A, B)** Graphics show IFN-γ, IL-17A, IL-10 and IL-4 concentrations in the supernatants of splenocyte cultures of vaccinated and non-vaccinated mice after being stimulated *ex vivo* with Hk PA14 (5×10^5^ CFU and 5×10^4^ CFU for 1:1 and 1:0.1 ratio of splenocytes:bacteria, respectively) for 3 days. No stimuli were added to control splenocytes. Mean ± SD. *P<0.05, (Mann-Whitney U test).
(JPG)

S14 Fig Analysis of myeloid cells in the lungs of IM-vaccinated mice. **(A, B)** Mice (*n* = 6/group) were immunized with α-PA vaccine (3×10^7^ CFU) or administered saline, according to the schedule; then lungs were collected. In **(B),** mice (*n* = 6/group) were infected with PA14 (1×10^6^ CFU), 9 h before the lungs were obtained. **(C, D)** Total number of cells in the lungs of non-infected **(C)** and infected **(D)** mice, detected by flow cytometry upon surface staining, according to the schedules indicated in **(A)** and **(B)**, respectively. Analysis regions were set according to FMO control-stained samples. Bars represent mean ± SD of data. *P<0.05 (t-test).
(JPG)

S15 Fig Lack of passively-transferred protection using α-PA immune sera. Mice survival after passive immunization with IgG-enriched mouse antiserum obtained from IN- and IM-immunized mice (live α-PA vaccine), or naïve serum and challenge with PA14 (3×10^6^ CFU) (*n* = 6–11 mice/group).
(JPG)

S16 Fig Different routes of immunization and impact on mice body weight. **(A)** Percentage of mice weight change after IN immunization with α-PA vaccine (2×10^8^ CFU, *n* = 8) and saline administration (*n* = 8). **(B)** Percentage of mice weight change after IM immunization with α-PA vaccine (3×10^7^ CFU, *n* = 7) and saline administration (*n* = 7). **(C)** Percentage of mice weight change after IN immunization with α-PA vaccine (3×10^7^ CFU, *n* = 9) and saline administration (*n* = 8). **(D)** Percentage of mice weight change after IN plus IM immunization with α-PA vaccine (3×10^7^ CFU, *n* = 9) and saline administration (*n* = 8). **(C, D)** The same control group–mice administered saline through IN plus IM route–was used for comparison. **(A-D)** Mean ± SD. *P<0.05 (pairwise comparison of weights within vaccinated mice, mixed ANOVA). ^#^*P*<0.05, (pairwise comparison of weights between groups, mixed ANOVA).
(JPG)

S17 Fig Safety and efficacy testing of IN immunization using low α-PA vaccine doses. **(A)** Mice were IN-immunized with α-PA vaccine (8×10^5^ CFU, *n* = 8; 7×10^6^ CFU, *n* = 8) or administered saline (*n* = 8), according to the schedule; then mice were challenged with PA14 (1×10^6^ CFU) on the day indicated. The MDSS system was applied for determining disease severity and surrogate endpoints, 16 hours after infection. Bacterial loads in the lungs were determined immediately after. **(B)** Percentage of mice weight change after IN immunization with α-PA vaccine or saline administration. Mean ± SD. *P<0.05, 7×10^6^ CFU-vaccinated group (pairwise comparison of weights within groups, mixed ANOVA). ^**#1**^*P*<0.05, saline *vs* 8×10^5^ CFU, 8×10^5^ *vs* 7×10^6^ CFU; ^**#2**^*P*<0.05, saline *vs* 7×10^6^ CFU, 8×10^5^ *vs* 7×10^6^ CFU (pairwise comparison of weights between groups, mixed ANOVA). **(C)** MDSS after immunization with the different doses of α-PA vaccine and challenge with PA14. **(D)** Bacterial loads in the lungs of mice after immunization with the different doses of α-PA vaccine and challenge with PA14.
(JPG)

S18 Fig Evaluation of live α-PA vaccine toxicity using ID and SC routes for immunization. **(A)** Percent of mice weight change after ID immunization with live α-PA vaccine (1.5×10^8^ CFU, *n* = 8; 3×10^7^ CFU, *n* = 9) or saline administration (*n* = 8). **(B)** Skin lesion produced by ID injection of live α-PA vaccine (1.5×10^8^ CFU). **(C)** Percent of mice weight change after SC immunization with live α-PA vaccine (1.5×10^8^ CFU, *n* = 8; 3×10^7^ CFU, *n* = 8) or saline administration (*n* = 8). **(D)** Skin lesion produced by SC injection of live α-PA vaccine (1.5×10^8^ CFU). **(A, C)** Mean ± SD.
(JPG)

S19 Fig Protective efficacy and systemic antibody responses generated by live α-PA vaccine administered ID and SC. **(A)** Immunization schedule using ID route, sampling of mice and lung infection challenge. **(B)** Mice survival after ID immunization with live α-PA vaccine (1.5×10^8^ CFU, *n* = 7; 3×10^7^ CFU, *n* = 9) or saline administration (*n* = 7) and challenge with PA14 (1×10^6^ CFU), causing acute pneumonia. **(C)** Titers of PAO1-specific antibodies after ID immunization with live α-PA vaccine (3×10^7^ CFU, *n* = 6; 1.5×10^8^ CFU, *n* = 7) or saline administration (*n* = 5). **(D)** Immunization schedule using SC route, sampling of mice and lung infection challenge. **(E)** Mice survival after SC immunization with live α-PA vaccine (1.5×10^8^ CFU, *n* = 8; 3×10^7^ CFU, *n* = 8) or saline administration (*n* = 8) and challenge with PA14 (1×10^6^ CFU), causing acute pneumonia. **(F)** Titers of PAO1-specific antibodies after SC immunization with live α-PA vaccine (3×10^7^ CFU, *n* = 7; 1.5×10^8^ CFU, *n* = 8) or saline administration (*n* = 8). **(B, E)** *P<0.05 (log-rank test), compared with saline group. **(C, F)** S, saline. *P<0.05 (Kruskal-Wallis test), compared with saline group.
(JPG)

S1 Table Strains used in the present work. (DOCX)

S2 Table Modified disease severity scoring system for monitoring of surrogate endpoints and assessment of disease severity in mouse pneumonia. (DOCX)
