# Supplementary material for: A live auxotrophic vaccine confers mucosal immunity and protection against lethal pneumonia caused by Pseudomonas aeruginosa
Source: PLoS Pathog. 2020 Feb 10;16(2):e1008311. doi: 10.1371/journal.ppat.1008311 (PMC7034913; doi:10.1371/journal.ppat.1008311)
Supplement: S1 Protocol — (DOCX) [file ppat.1008311.s001.docx]

**S1 Protocol. Flow cytometric analysis**

The assessment of cell surface lineage or activation markers on different splenic and lung leukocyte populations was performed by flow cytometric analysis (fluorescence-activated cell sorter [FACS] analysis). Spleens and lungs were aseptically removed, homogenized to single-cell suspensions in RPMI-1640 complete medium (RPMI-1640 supplemented with 10 mM Hepes, 200 IU/mL penicillin, 200 μg/mL streptomycin, 50 μM 2-mercaptoethanol, and 10% FBS (all from Sigma-Aldrich) and, when necessary, red blood cells were lysed. Lung cells were incubated in 2 mg/mL Collagenase D (Roche) for 45 min with 5% CO_2_ at 37 ºC before homogenization. 10^6^ cells were used per sample. Prior to antibody staining, cells were washed in PBS and incubated with a Fixable Viability Dye (FVD; eBioscience) – Allophycocyanin-eFluor 780 (APC-eF780)-conjugate to allow dead cell exclusion. The following mAb were used (at previously determined optimal dilutions) for surface antigen staining after pre-incubation with purified anti-mouse CD16/CD32 (BD biosciences) for FcγR blocking: anti-mouse CD3 Brilliant Violet 510 (BV510)-conjugate (clone 17A2), anti-mouse F4/80 Phycoerythrin (PE)-conjugate (clone BM8), anti-mouse I-A/I-E Peridinin Chlorophyll Protein Complex (PerCP)-conjugate (clone M5/114.15.2), anti-mouse CD45 PerCP/Cy5.5-conjugate (clone I3/2.3) or anti-mouse CD45 FITC-conjugate (clone 30-F11), anti-mouse CD127 PE-cychrome 7 (PE-Cy7)-conjugate (clone A7R34), anti-mouse CD11c BV421-conjugate (clone N418), and anti-mouse Ly6G Alexa Fluor 647 (AF647)-conjugate (clone 1A8) (all from BioLegend); anti-mouse CD44 PerCP/Cyanine 5.5 (PerCP/Cy5.5)-conjugate (clone IM7; eBioscience); anti-mouse gamma delta T-cell Receptor FITC-conjugate (clone GL3), anti-mouse CD62L (PE)-conjugate (clone MEL-14), anti-mouse CD4 Fluorescein isothiocyanate (FITC)- or BD Horizon V450 (V450)-conjugate (clone RM4–5), anti-mouse CD11b BV510-conjugate (clone M1/70), and anti-mouse Ly6C PE-Cy7-conjugate (clone AL-21) (all from BD Biosciences). For intracellular cytokine detection, cells were counted and plated in round bottom 96 plates, at a concentration of 5 × 10^6^ cells/mL in complete RMPI-1640 medium. Cells were incubated in a humidified atmosphere with 5% CO_2_ at 37 °C for 4 h under stimulation with 10 ng/mL phorbol myristate acetate (PMA) and 1 μg/mL ionomycin, in the presence of 10 μg/mL Brefeldin A (all from Sigma). Upon incubation with the different stimuli, cells were recovered, stained with FVD-APC eF780-conjugate and incubated with anti-mouse CD16/CD32, prior to staining with anti-CD3 and anti-CD4 or anti-gamma delta TCR mAbs. Following extracellular staining the cells were washed, fixed with 2% formaldehyde in PBS, and permeabilized with 0.05% saponin (Sigma) in PBS solution. Intracytoplasmic staining was carried out with anti-IFN-γ FITC- or APC-conjugate (clone XMG1.2), anti-mouse IL-4 PE/Cy7-conjugate (clone BVD4-1D11), and anti-IL-10 PE-conjugate (clone JESS-16E3) (all from BD Biosciences), anti-IL-17A FITC or PE-conjugate (clone TC11-18H10.1) and anti-TNF-α PerCP/Cy5.5-conjugate (clone MP6-XT22) (all from BioLegend) mAb after FcγR blocking. Immunofluorescence cytometric analysis was performed with a FACSCanto II system (BD Biosciences) using the FACSDiva software (BD) and compensated and analysed in FlowJo version 10.4.2. (Tree Star). A minimum of 150,000 events were acquired per sample. A biexponential transformation was applied to improve data visualization. Fluorescence minus one (FMO) gating was used to define gates. Isotype controls were used to evaluate unspecific staining.
