# Supplementary figures and images for: A live auxotrophic vaccine confers mucosal immunity and protection against lethal pneumonia caused by Pseudomonas aeruginosa
Source: PLoS Pathog. 2020 Feb 10;16(2):e1008311. doi: 10.1371/journal.ppat.1008311 (PMC7034913; doi:10.1371/journal.ppat.1008311)

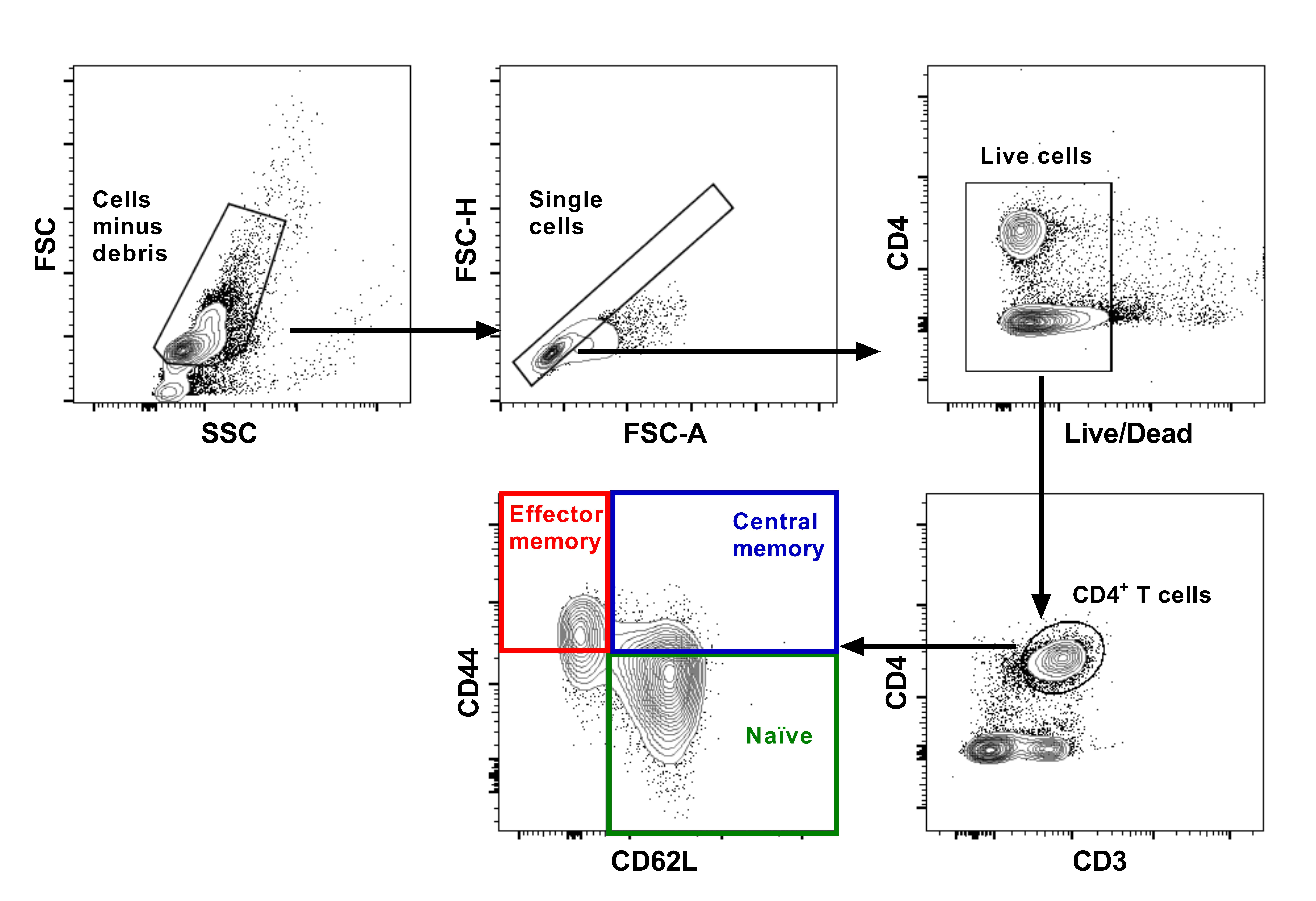

Supplement: S1 Fig — Debris were excluded (Cell minus debris) based on FSC and SSC parameters and doublets were excluded (Single cells) in FSC-H versus FSC-A plots. Viable cells (Live cells) were gated as fixable viability dye negative cells. CD4 T cells were defined as CD3+CD4+ and quadrants were set, based on FMO stainings on contour plots, to define naïve (CD62L+CD44-), central memory (CD62L+CD44+) and effector memory (CD62L-CD44+) cells. (JPG) [file ppat.1008311.s002.jpg]

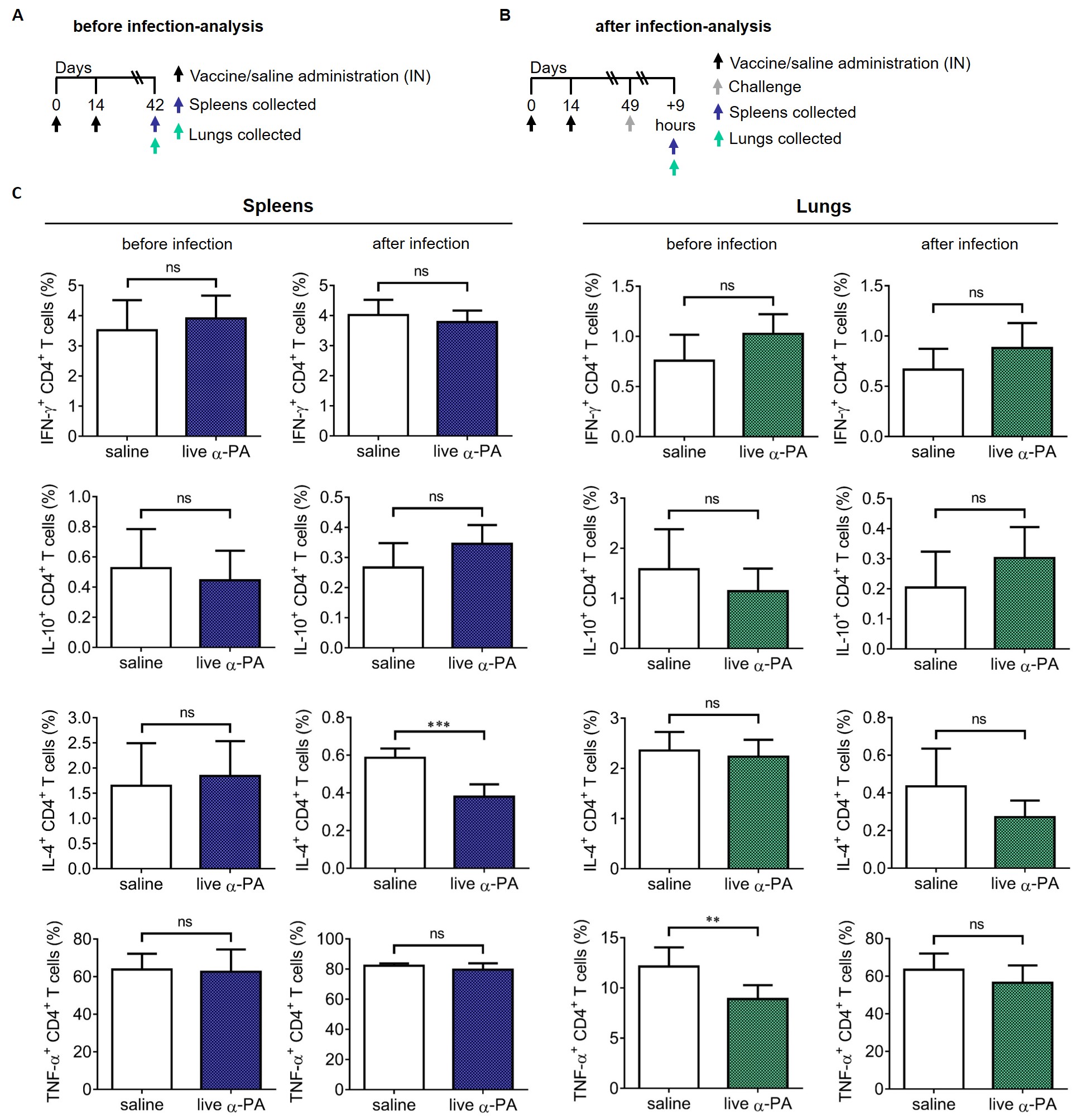

Supplement: S2 Fig — (A) BALB/c mice (n = 6/group) were immunized with live α-PA vaccine (2×10^8^ CFU) or administered saline, according to the schedule; then spleens and lungs were collected on the day indicated. (B) BALB/c mice (n = 6/group) were immunized with live α-PA vaccine (2×10^8^ CFU) or administered saline, according to the schedule; then mice were infected with PA14 (1×10^6^ CFU) on the day indicated. Spleens and lungs were collected 9 hours after. (C) Frequency (percentage) of splenic and lung CD4-gated T cells expressing IFN-γ, IL-10, IL-4 and TNF-α of infected and non-infected mice, detected by intracellular staining after stimulation with PMA/ionomycin. Bars represent mean ± SD of data. *P<0.05, **P<0.01, ***P<0.001 (t-test), compared with saline group. ns, not significant. (JPG) [file ppat.1008311.s003.jpg]

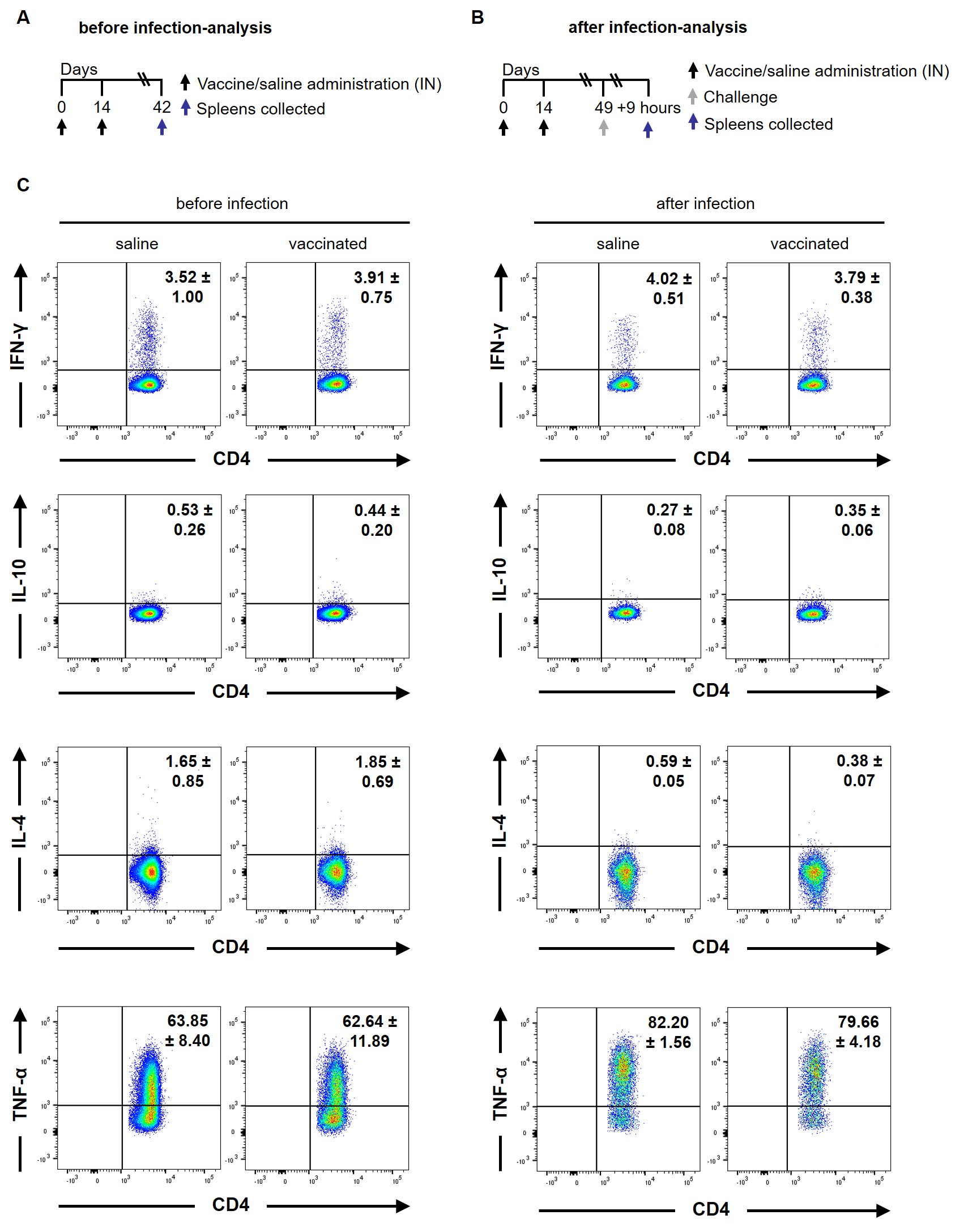

Supplement: S3 Fig — (A) BALB/c mice (n = 6/group) were immunized with live α-PA vaccine (2×10^8^ CFU) or administered saline, according to the schedule; then spleens were collected on the day indicated. (B) BALB/c mice (n = 6/group) were immunized with live α-PA vaccine (2×10^8^ CFU) or administered saline, according to the schedule; then mice were infected with PA14 (1×10^6^ CFU) on the day indicated. Spleens were collected 9 hours after. (C) Representative examples of CD4-gated T cells of spleens obtained from infected and non-infected mice, detected by intracellular staining, after stimulation with PMA/ionomycin. Analysis regions were set according to FMO and isotype control-stained samples. Numbers inside dot plot regions represent means ± SD of the frequency of cells due to respective cytokine staining. (JPG) [file ppat.1008311.s004.jpg]

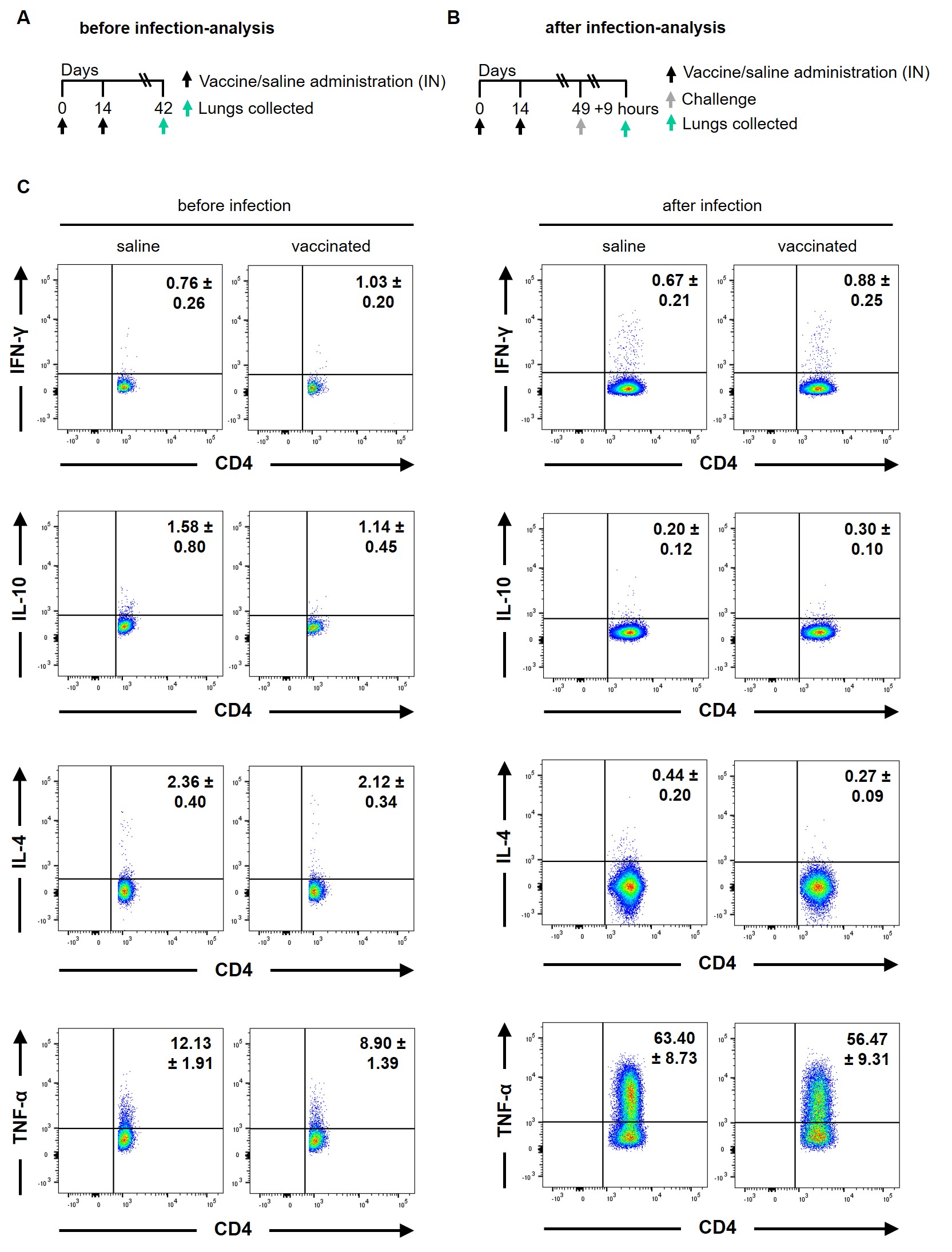

Supplement: S4 Fig — (A) BALB/c mice (n = 6/group) were immunized with live α-PA vaccine (2×10^8^ CFU) or administered saline, according to the schedule; then lungs were collected on the day indicated. (B) BALB/c mice (n = 6/group) were immunized with live α-PA vaccine (2×10^8^ CFU) or administered saline, according to the schedule; then mice were infected with PA14 (1×10^6^ CFU) on the day indicated. Lungs were collected 9 hours after. (C) Representative examples of CD4-gated T cells of lungs obtained from infected and non-infected mice, detected by intracellular staining, after stimulation with PMA/ionomycin. Analysis regions were set according to FMO and isotype control-stained samples. Numbers inside dot plot regions represent means ± SD of the frequency of cells due to respective cytokine staining. (JPG) [file ppat.1008311.s005.jpg]

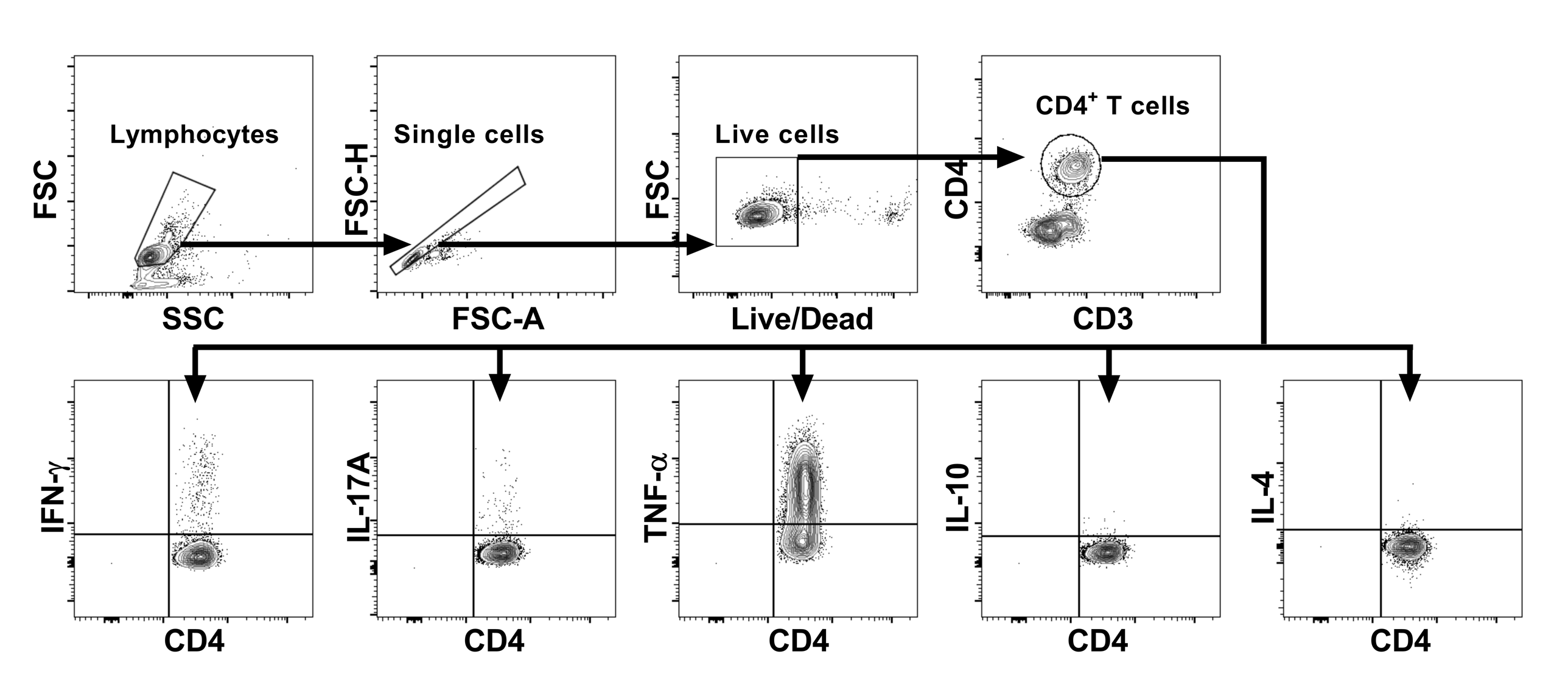

Supplement: S5 Fig — Cells were stimulated with PMA/ionomycin in the presence of Brefeldin A for 4 h, followed by lineage marker surface staining. Produced cytokines were labelled by intracellular staining. Lymphocytes were selected based on FSC and SSC parameters, doublets were then excluded (Single cells) and viable cells (Live cells) were gated as fixable viability dye negative cells. CD4 T cells were defined as CD3+CD4+ and quadrants were set, based on FMO staining on contour plots, to define cytokine-producing cells. Representative contour plots of splenic CD4 T cells producing IFN-γ, IL-17A, IL-10, and IL-4, are shown. (JPG) [file ppat.1008311.s006.jpg]

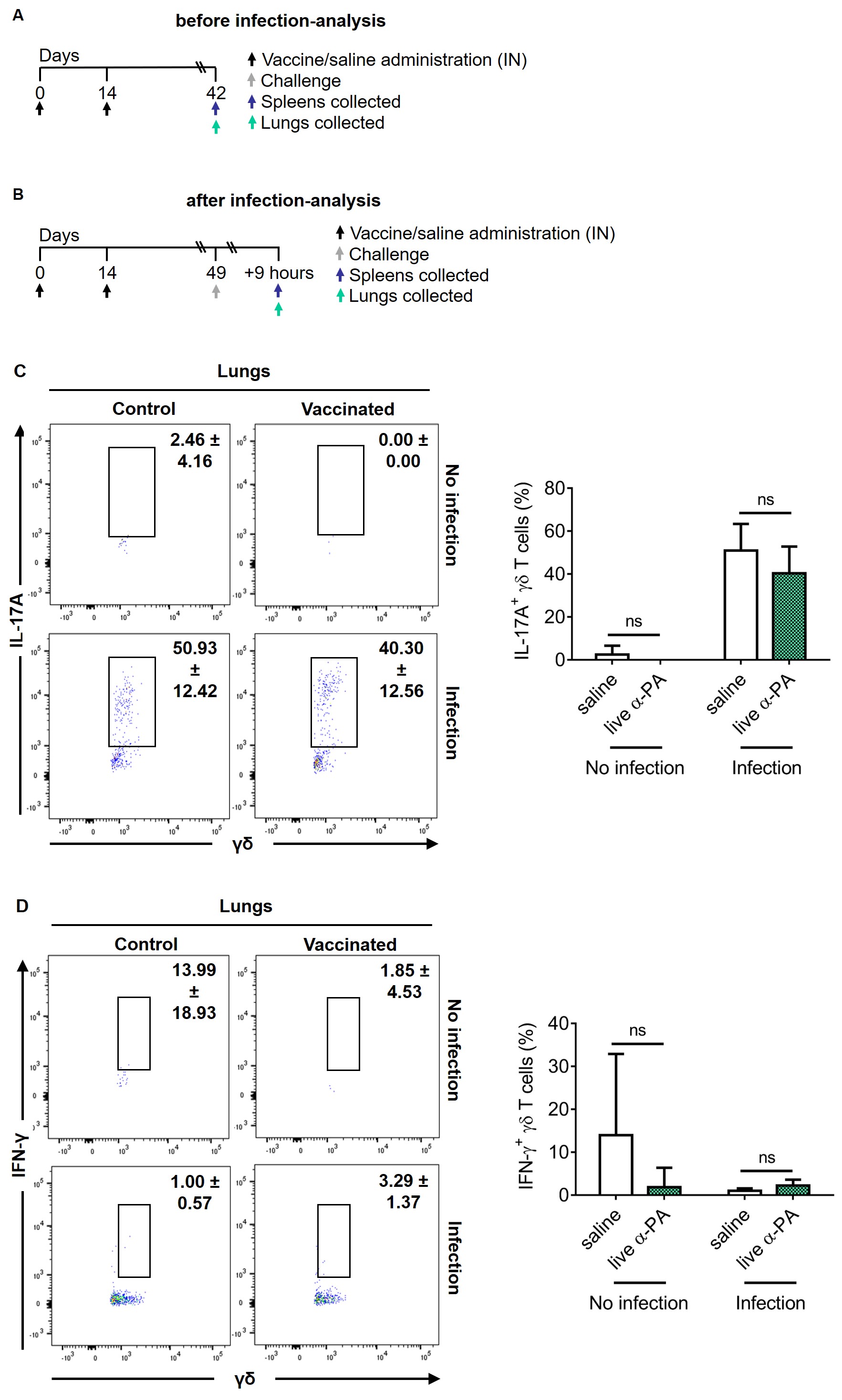

Supplement: S6 Fig — (A) BALB/c mice (n = 6/group) were immunized with live α-PA vaccine (2×10^8^ CFU) or administered saline, according to the schedule; then spleens and lungs were collected on the day indicated. (B) BALB/c mice (n = 6/group) were immunized with live α-PA vaccine (2×10^8^ CFU) or administered saline, according to the schedule; then mice were infected with PA14 (1×10^6^ CFU) on the day indicated. Spleens and lungs were collected 9 hours after. (C, D) Representative examples and frequency (percentage) of lung γδ-gated T cells expressing IL-17A (C) and IFN-γ (D) of infected and non-infected mice, detected by intracellular staining after stimulation for 4 h with PMA/ionomycin in the presence of Brefeldin A. Analysis regions were set according to FMO and isotype control-stained samples. Numbers inside dot plot regions represent means ± SD of the frequency of cells due to respective cytokine staining. Bars represent mean ± SD of data. *P<0.05, **P<0.01 (t-test), compared with saline group. (JPG) [file ppat.1008311.s007.jpg]

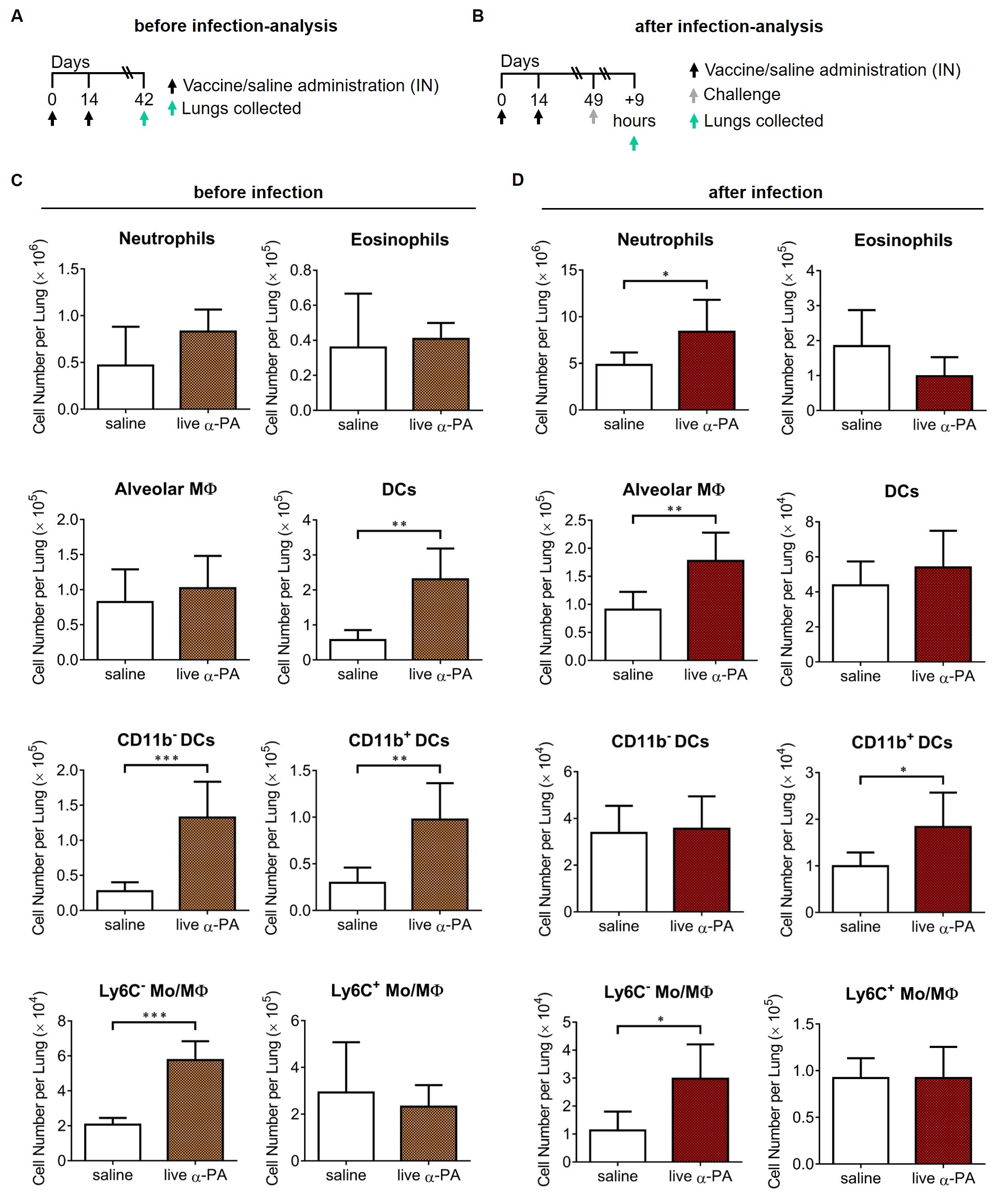

Supplement: S9 Fig — (A, B) Mice (n = 6/group) were immunized with α-PA vaccine (2×10^8^ CFU) or administered saline, according to the schedule; then lungs were collected. In (B), mice (n = 6/group) were infected with PA14 (1×10^6^ CFU), 9 h before the lungs were obtained. (C, D) Total number of cells in the lungs of non-infected (C) and infected (D) mice, detected by flow cytometry upon surface staining, according to the schedules indicated in (A) and (B), respectively. Analysis regions were set according to FMO control-stained samples. Bars represent mean ± SD of data. *P<0.05, **P<0.01, ***P<0.001, ****P<0.0001 (t-test). (JPG) [file ppat.1008311.s010.jpg]

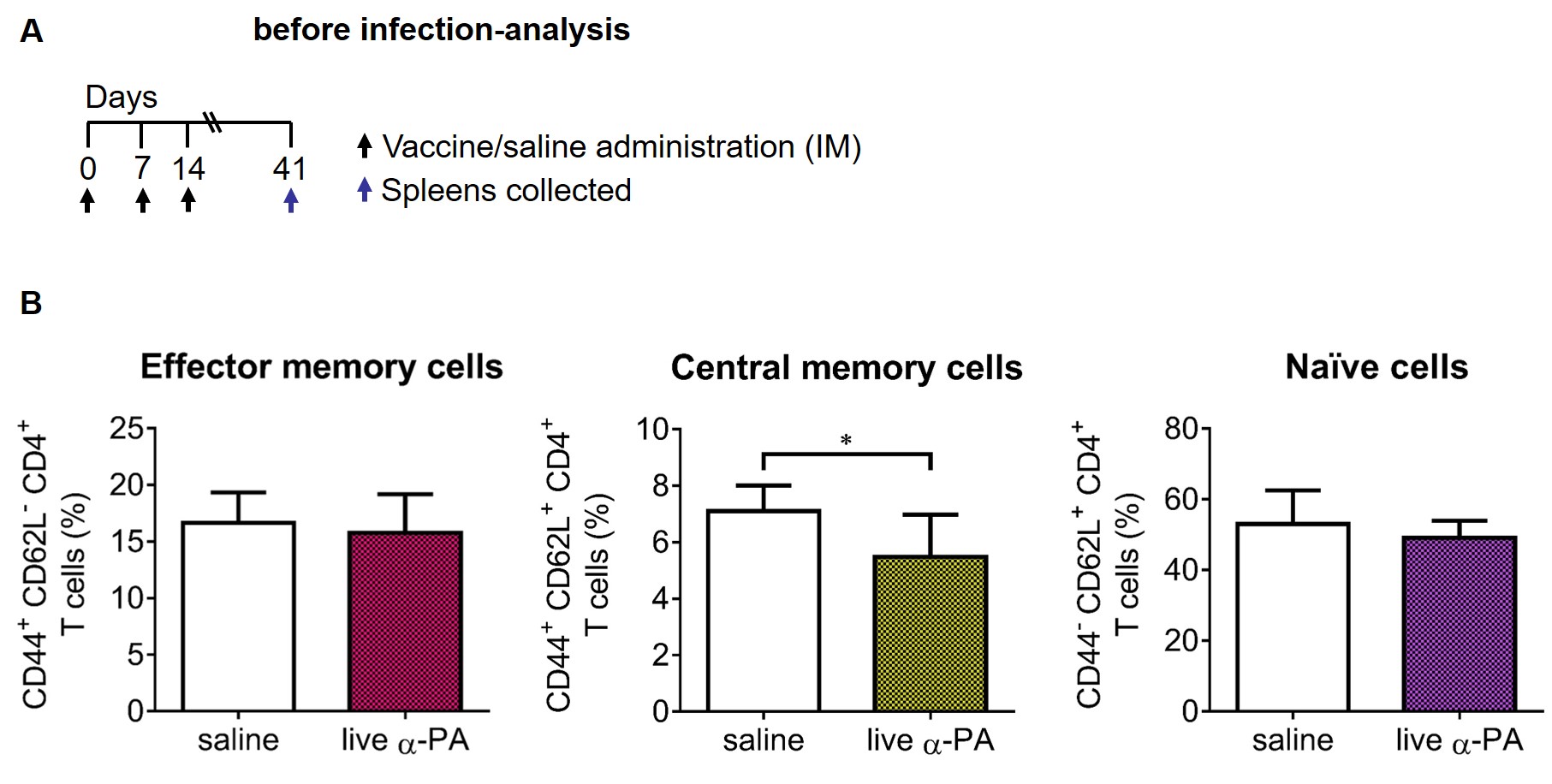

Supplement: S11 Fig — (A) Mice (n = 6/group) were immunized with α-PA vaccine (3×10^7^ CFU) or administered saline, according to the schedule; then spleens were collected. (B) Percentage of spleen effector memory (CD44+ CD62L-), central memory (CD44+ CD62L+) and naïve (CD44- CD62L+) CD4+ T cells. Mean ± SD of data. *P<0.05 (t-test). (JPG) [file ppat.1008311.s012.jpg]

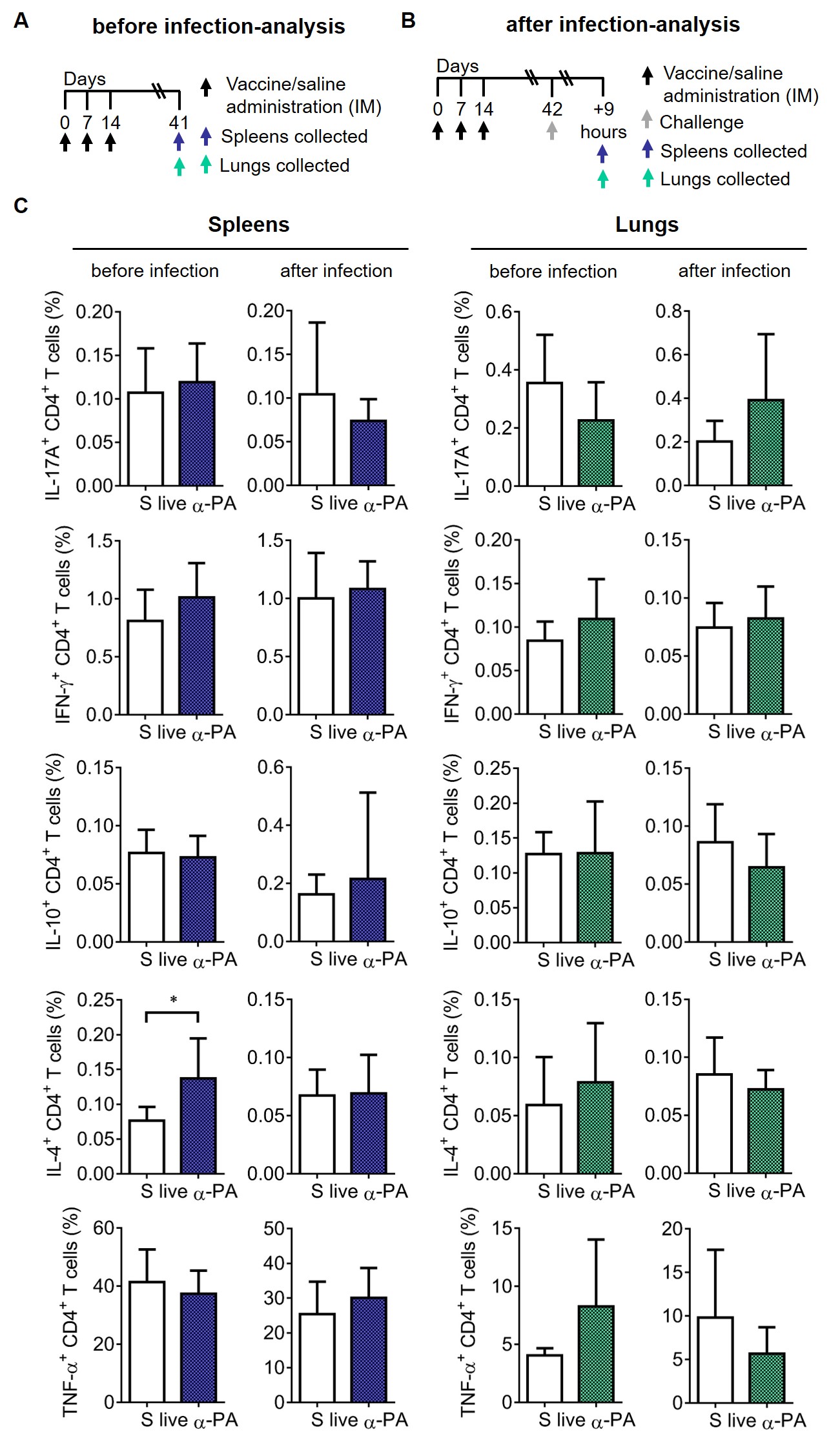

Supplement: S12 Fig — (A) BALB/c mice (n = 6/group) were immunized with live α-PA vaccine (3×10^7^ CFU) or administered saline, according to the schedule; then spleens and lungs were collected on the day indicated. (B) BALB/c mice (n = 6/group) were immunized with live α-PA vaccine (3×10^7^ CFU) or administered saline, according to the schedule; then mice were infected with PA14 (1×10^6^ CFU) on the day indicated. Spleens and lungs were collected 9 hours after. (C) Frequency (percentage) of splenic and lung CD4-gated T cells expressing IL-17A, IFN-γ, IL-10, IL-4 and TNF-α of infected and non-infected mice, detected by intracellular staining after stimulation with PMA/ionomycin. Bars represent mean ± SD of data. *P<0.05 (t-test). (JPG) [file ppat.1008311.s013.jpg]

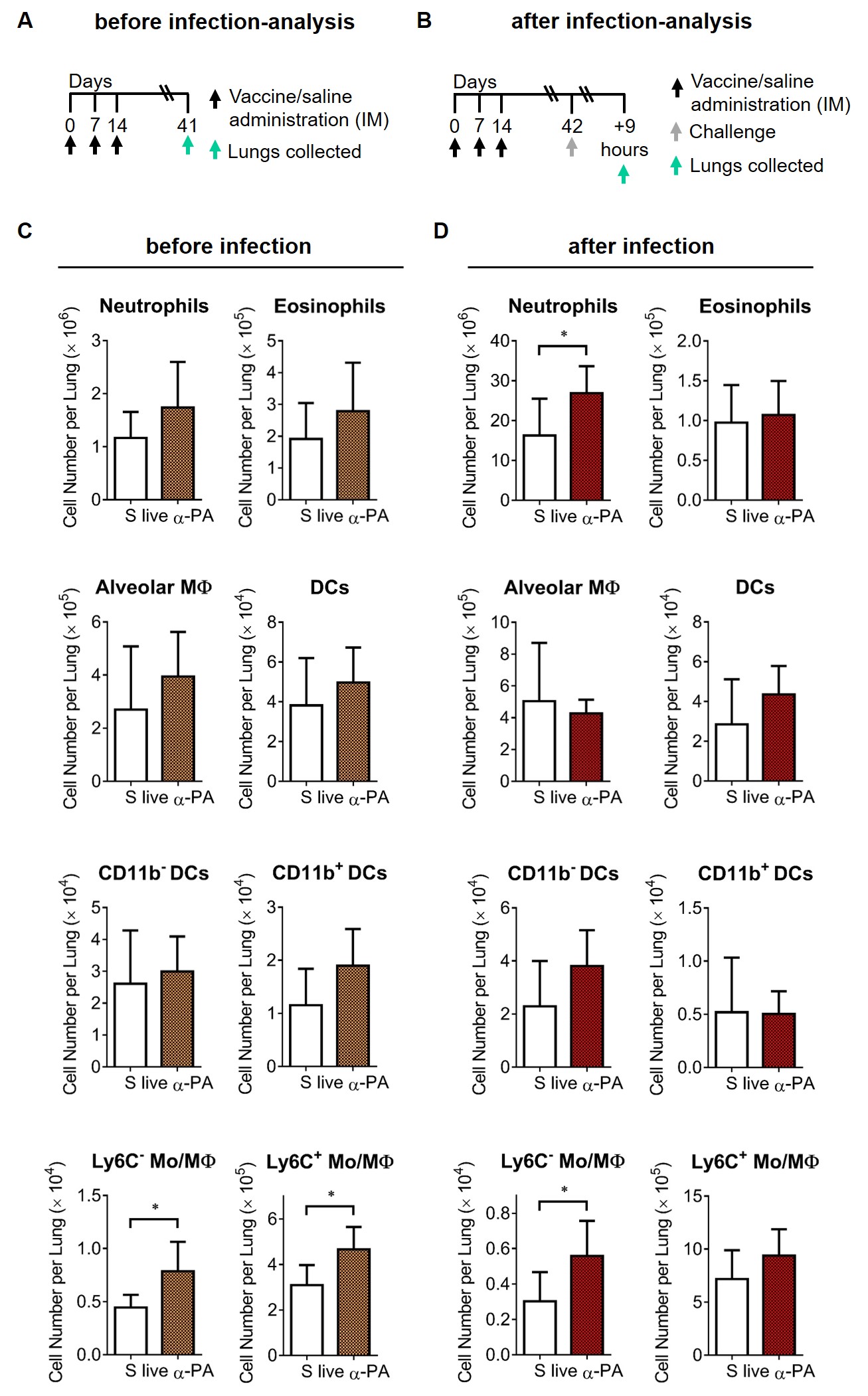

Supplement: S14 Fig — (A, B) Mice (n = 6/group) were immunized with α-PA vaccine (3×10^7^ CFU) or administered saline, according to the schedule; then lungs were collected. In (B), mice (n = 6/group) were infected with PA14 (1×10^6^ CFU), 9 h before the lungs were obtained. (C, D) Total number of cells in the lungs of non-infected (C) and infected (D) mice, detected by flow cytometry upon surface staining, according to the schedules indicated in (A) and (B), respectively. Analysis regions were set according to FMO control-stained samples. Bars represent mean ± SD of data. *P<0.05 (t-test). (JPG) [file ppat.1008311.s015.jpg]

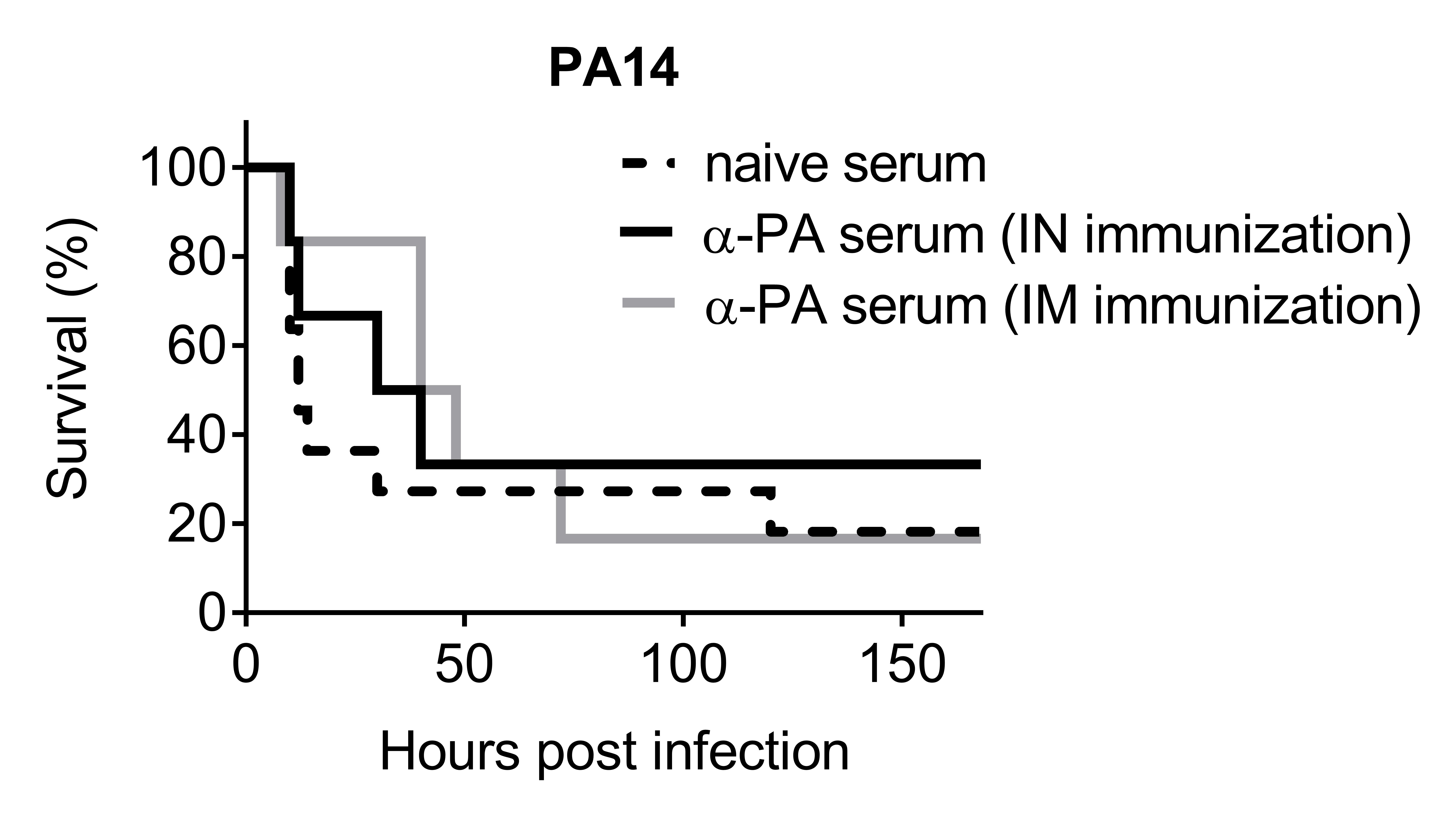

Supplement: S15 Fig — Mice survival after passive immunization with IgG-enriched mouse antiserum obtained from IN- and IM-immunized mice (live α-PA vaccine), or naïve serum and challenge with PA14 (3×10^6^ CFU) (n = 6–11 mice/group). (JPG) [file ppat.1008311.s016.jpg]

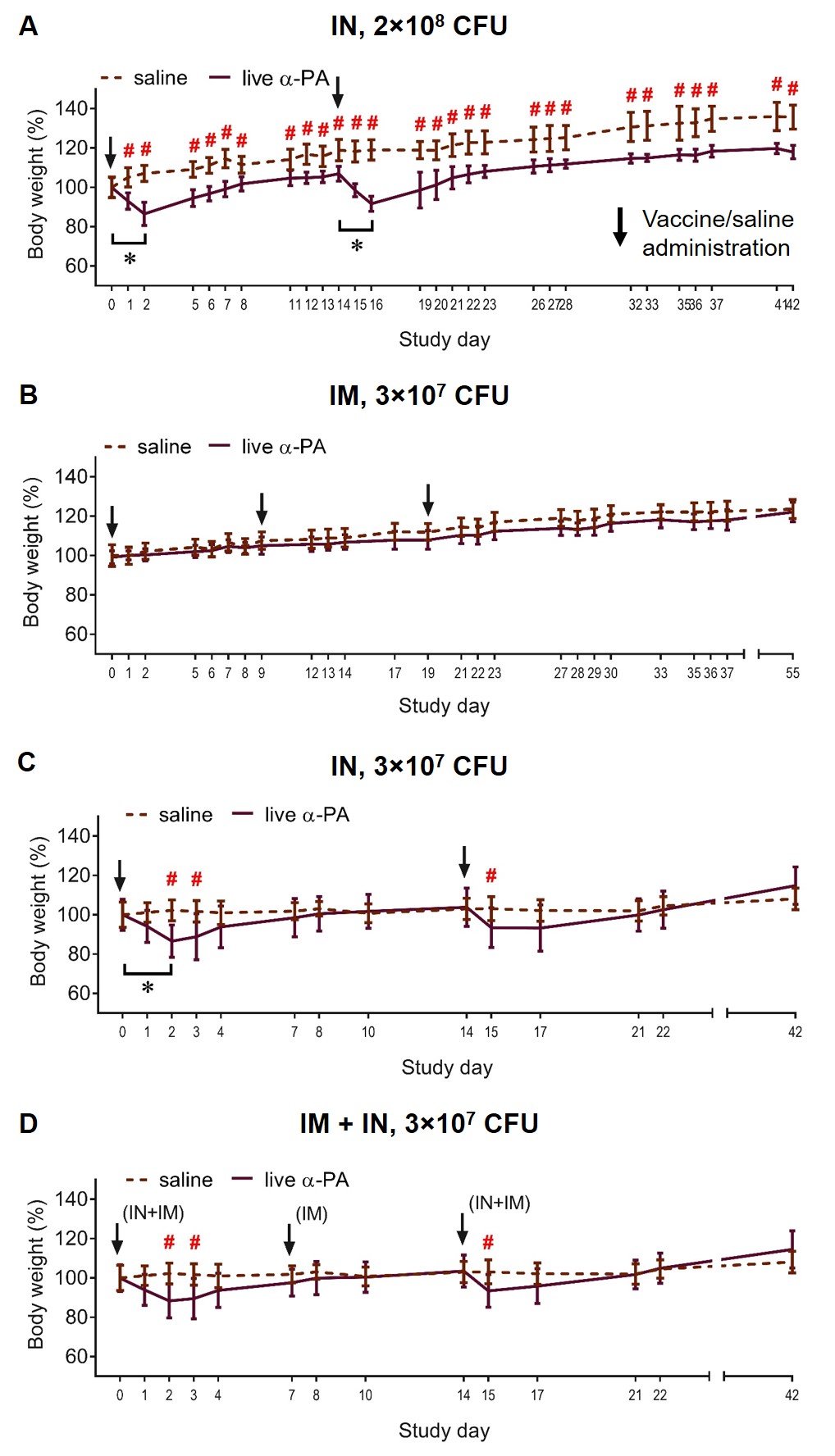

Supplement: S16 Fig — (A) Percentage of mice weight change after IN immunization with α-PA vaccine (2×10^8^ CFU, n = 8) and saline administration (n = 8). (B) Percentage of mice weight change after IM immunization with α-PA vaccine (3×10^7^ CFU, n = 7) and saline administration (n = 7). (C) Percentage of mice weight change after IN immunization with α-PA vaccine (3×10^7^ CFU, n = 9) and saline administration (n = 8). (D) Percentage of mice weight change after IN plus IM immunization with α-PA vaccine (3×10^7^ CFU, n = 9) and saline administration (n = 8). (C, D) The same control group–mice administered saline through IN plus IM route–was used for comparison. (A-D) Mean ± SD. *P<0.05 (pairwise comparison of weights within vaccinated mice, mixed ANOVA). #P<0.05, (pairwise comparison of weights between groups, mixed ANOVA). (JPG) [file ppat.1008311.s017.jpg]

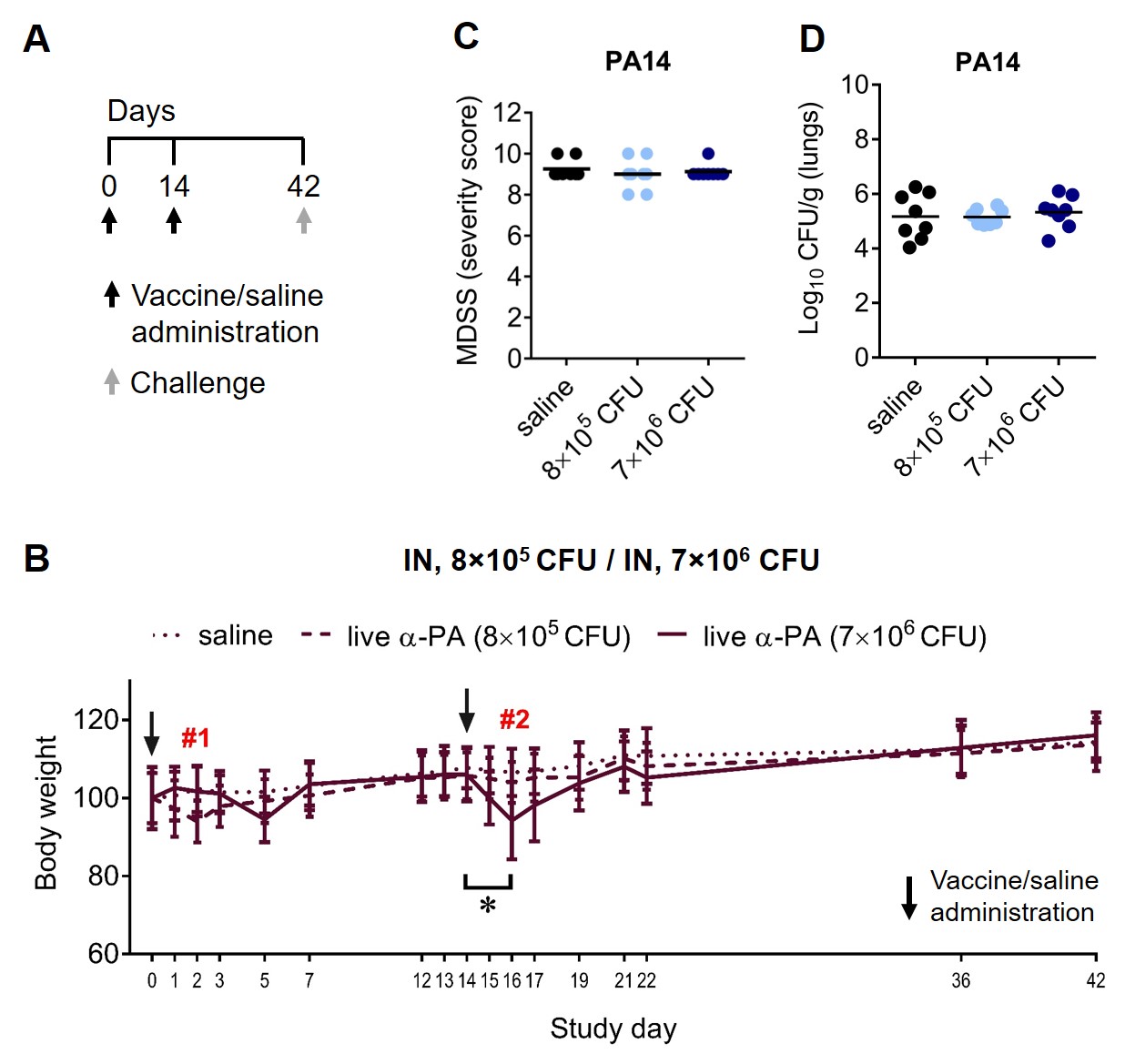

Supplement: S17 Fig — (A) Mice were IN-immunized with α-PA vaccine (8×10^5^ CFU, n = 8; 7×10^6^ CFU, n = 8) or administered saline (n = 8), according to the schedule; then mice were challenged with PA14 (1×10^6^ CFU) on the day indicated. The MDSS system was applied for determining disease severity and surrogate endpoints, 16 hours after infection. Bacterial loads in the lungs were determined immediately after. (B) Percentage of mice weight change after IN immunization with α-PA vaccine or saline administration. Mean ± SD. *P<0.05, 7×10^6^ CFU-vaccinated group (pairwise comparison of weights within groups, mixed ANOVA). #1P<0.05, saline vs 8×10^5^ CFU, 8×10^5^ vs 7×10^6^ CFU; #2P<0.05, saline vs 7×10^6^ CFU, 8×10^5^ vs 7×10^6^ CFU (pairwise comparison of weights between groups, mixed ANOVA). (C) MDSS after immunization with the different doses of α-PA vaccine and challenge with PA14. (D) Bacterial loads in the lungs of mice after immunization with the different doses of α-PA vaccine and challenge with PA14. (JPG) [file ppat.1008311.s018.jpg]

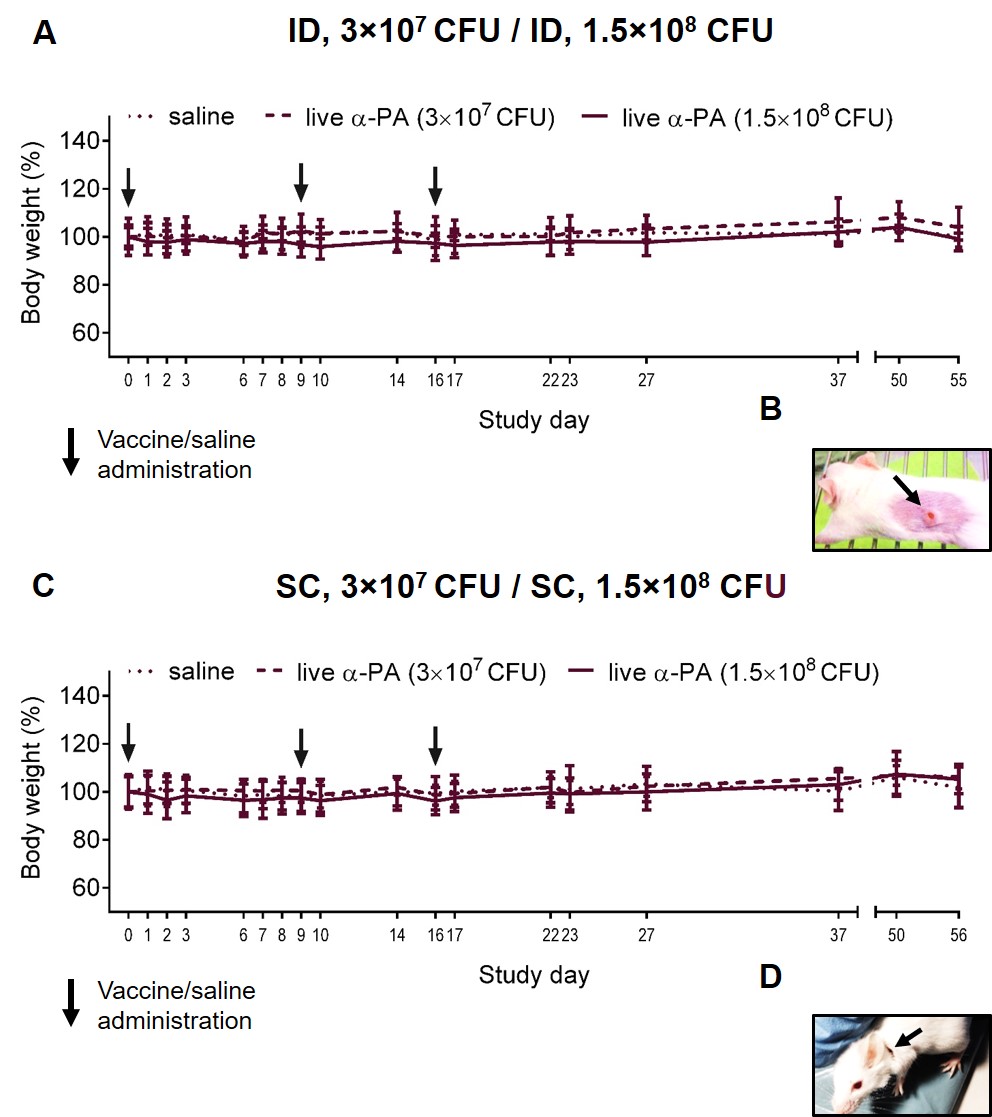

Supplement: S18 Fig — (A) Percent of mice weight change after ID immunization with live α-PA vaccine (1.5×10^8^ CFU, n = 8; 3×10^7^ CFU, n = 9) or saline administration (n = 8). (B) Skin lesion produced by ID injection of live α-PA vaccine (1.5×10^8^ CFU). (C) Percent of mice weight change after SC immunization with live α-PA vaccine (1.5×10^8^ CFU, n = 8; 3×10^7^ CFU, n = 8) or saline administration (n = 8). (D) Skin lesion produced by SC injection of live α-PA vaccine (1.5×10^8^ CFU). (A, C) Mean ± SD. (JPG) [file ppat.1008311.s019.jpg]

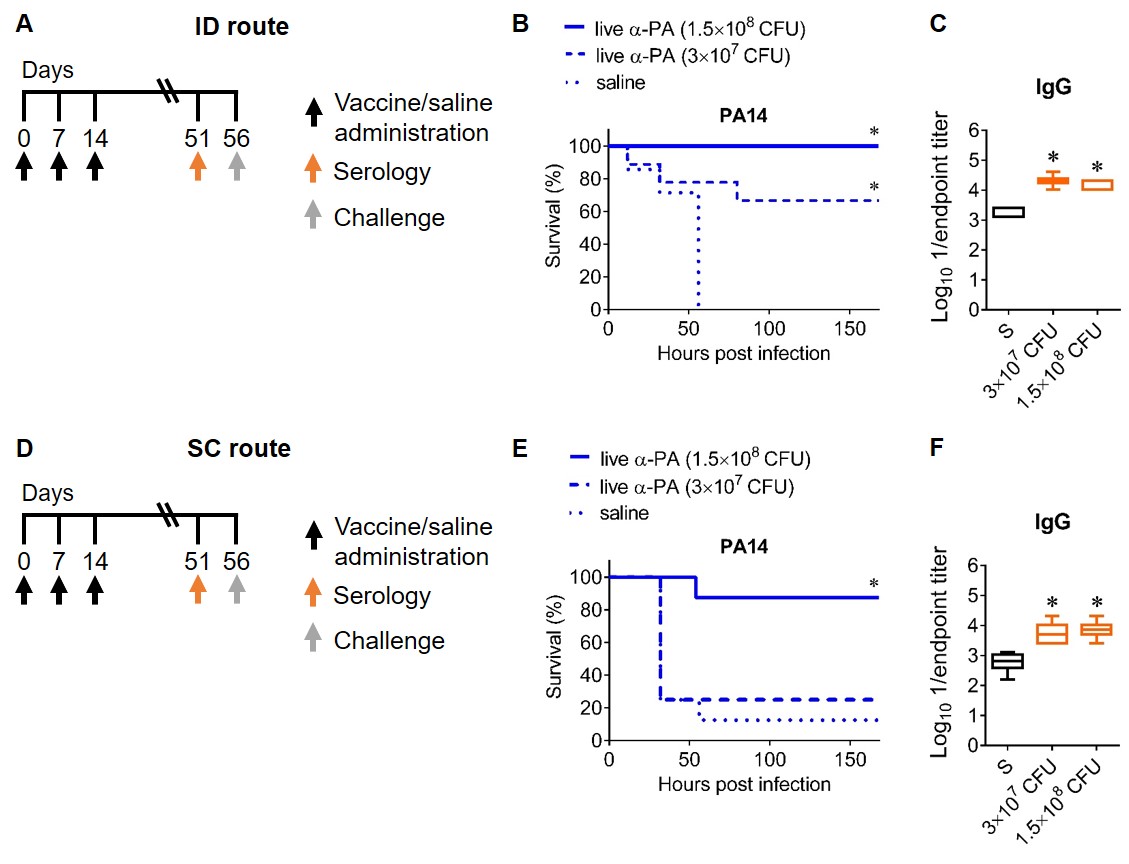

Supplement: S19 Fig — (A) Immunization schedule using ID route, sampling of mice and lung infection challenge. (B) Mice survival after ID immunization with live α-PA vaccine (1.5×10^8^ CFU, n = 7; 3×10^7^ CFU, n = 9) or saline administration (n = 7) and challenge with PA14 (1×10^6^ CFU), causing acute pneumonia. (C) Titers of PAO1-specific antibodies after ID immunization with live α-PA vaccine (3×10^7^ CFU, n = 6; 1.5×10^8^ CFU, n = 7) or saline administration (n = 5). (D) Immunization schedule using SC route, sampling of mice and lung infection challenge. (E) Mice survival after SC immunization with live α-PA vaccine (1.5×10^8^ CFU, n = 8; 3×10^7^ CFU, n = 8) or saline administration (n = 8) and challenge with PA14 (1×10^6^ CFU), causing acute pneumonia. (F) Titers of PAO1-specific antibodies after SC immunization with live α-PA vaccine (3×10^7^ CFU, n = 7; 1.5×10^8^ CFU, n = 8) or saline administration (n = 8). (B, E) *P<0.05 (log-rank test), compared with saline group. (C, F) S, saline. *P<0.05 (Kruskal-Wallis test), compared with saline group. (JPG) [file ppat.1008311.s020.jpg]
