# Supplementary material for: A live auxotrophic vaccine confers mucosal immunity and protection against lethal pneumonia caused by Pseudomonas aeruginosa
Source: PLoS Pathog. 2020 Feb 10;16(2):e1008311. doi: 10.1371/journal.ppat.1008311 (PMC7034913; doi:10.1371/journal.ppat.1008311)
Supplement: S1 Table — (DOCX) [file ppat.1008311.s021.docx]

**S1 Table. Strains used in the present work.**

| *P. aeruginosa* strain | Relevant features | Source or reference |
| --- | --- | --- |
| PAO1 | Reference strain; exoS^+^/exoU^-^ genotype | CECT, [1] |
| PAO1 Δ*murI* | PAO1 derivative; ΔPA4662 | [2] |
| PAO1 ExoU^+^ | PAO1 derivative expressing and secreting ExoU; Cb^R^ | [3] |
| PA14 | Reference strain; hypervirulent; exoS^-^/exoU^+^ genotype | [1,4] |
| ST235 | Clinical strain; epidemic clone; XDR; exoS^-^/exoU^+^ genotype | [1,5] |
| ST175 | Clinical strain; epidemic clone; XDR; exoS^+^/exoU^-^ genotype | [1,5,6] |
| LES400 | Liverpool epidemic strain from a cystic fibrosis patient with chronic infection | [7] |
| 12142 | Liverpool epidemic strain isolate from cystic fibrosis patient | [8] |
| 51442390 | A Coruña Hospital isolate from cystic fibrosis patient; mucoid phenotype; Mem^R^ | Laboratory collection |
| 29606 | A Coruña Hospital isolate from cystic fibrosis patient; mucoid phenotype | Laboratory collection |
| 28757 | A Coruña Hospital isolate from cystic fibrosis patient; mucoid phenotype | Laboratory collection |
| LES431 | Liverpool epidemic strain from a non-cystic fibrosis parent with pneumonia | [7] |
| 51441321 | A Coruña Hospital isolate from bronchiectasis patient; Mem^R^, Fep^R^ | Laboratory collection |
| 28562 | A Coruña Hospital isolate from bronchiectasis patient; mucoid phenotype; | Laboratory collection |

**References**

1. Gómez-Zorrilla S, Juan C, Cabot G, Camoez M, Tubau F, Oliver A, et al. Impact of multidrug resistance on the pathogenicity of Pseudomonas aeruginosa: in vitro and in vivo studies. Int J Antimicrob Agents. 2016;47(5):368–74.

2. Cabral MP, García P, Beceiro A, Rumbo C, Pérez A, Moscoso M, et al. Design of live attenuated bacterial vaccines based on D-glutamate auxotrophy. Nat Commun. 2017 May 26;8:15480.

3. Allewelt M, Coleman FT, Grout M, Priebe GP, Pier GB. Acquisition of expression of the Pseudomonas aeruginosa ExoU cytotoxin leads to increased bacterial virulence in a murine model of acute pneumonia and systemic spread. Infect Immun. 2000 Jul;68(7):3998–4004.

4. Lee DG, Urbach JM, Wu G, Liberati NT, Feinbaum RL, Miyata S, et al. Genomic analysis reveals that Pseudomonas aeruginosa virulence is combinatorial. Genome Biol. 2006 Jan;7(10):R90.

5. Cabot G, Ocampo-Sosa AA, Domínguez MA, Gago JF, Juan C, Tubau F, et al. Genetic markers of widespread extensively drug-resistant Pseudomonas aeruginosa high-risk clones. Antimicrob Agents Chemother. 2012 Dec;56(12):6349–57.

6. Viedma E, Juan C, Otero JR, Oliver A, Chaves F. Draft Genome Sequence of VIM-2-Producing Multidrug-Resistant Pseudomonas aeruginosa ST175, an Epidemic High-Risk Clone. Genome Announc. 2013 Jan;1(2):e0011213.

7. Salunkhe P, Smart CHM, Morgan AW, Panagea S, Walshaw MJ, Hart A, et al. A Cystic Fibrosis Epidemic Strain of. J Bacteriol. 2005;187(14):4908–20.

8. Tomás M, Doumith M, Warner M, Turton JF, Beceiro A, Bou G, et al. Efflux pumps, OprD porin, AmpC β-lactamase, and multiresistance in Pseudomonas aeruginosa isolates from cystic fibrosis patients. Antimicrob Agents Chemother. 2010;54(5):2219–24.
