# Supplementary material for: A live auxotrophic vaccine confers mucosal immunity and protection against lethal pneumonia caused by Pseudomonas aeruginosa
Source: PLoS Pathog. 2020 Feb 10;16(2):e1008311. doi: 10.1371/journal.ppat.1008311 (PMC7034913; doi:10.1371/journal.ppat.1008311)
Supplement: S2 Table — (DOCX) [file ppat.1008311.s022.docx]

**S2 Table. Modified disease severity scoring system for monitoring of surrogate endpoints and assessment of disease severity in mouse pneumonia.**

| **Murine disease severity score (MDSS)** | | | | | |
| --- | --- | --- | --- | --- | --- |
| **Score** | **0** | **1** | **2** | **3** | **HEP** |
| **Body Weight** | No changes | BW loss 1-6% | BW loss 7-14% | BW loss 15-19% | BW loss >20% |
| **Appearance** | Normal | General lack of grooming | Piloerection, ocular and nasal discharges, hunched up | Above and eyes half close | - |
| **Behavior** | Normal | Minor changes or exaggerated response when provoked | Less mobile/isolated but alert | Restless or still, not alert | - |
| **Hydration** | Normal | - | - | Abnormal skin test pinch | - |
| **Respiratory movement** | Normal | - | - | Dyspnea | - |

BW: body weight; HEP: human endpoint.
